# Alcohol consumption in metabolic dysfunction-associated steatotic liver disease (MASLD): understanding the gut–liver crosstalk for clinical translation

**DOI:** 10.1080/19490976.2026.2631834

**Published:** 2026-02-21

**Authors:** Raquel Benedé-Ubieto, Olga Estévez-Vázquez, Rana Acar, Hector Leal-Lassalle, Alejandro H. Gutierrez, Ana Redondo-Urzainqui, Salvador Iborra, Vera E. Odintsova, Alexander Tyakht, José María Herranz, Zehra Firat, Merve Basol, Busra Korkmaz, Carlos Sanz-García, Oriol Juanola, Esther Caparrós, Rubén Francés, Andreea Ciudin, Juan M. Pericàs, Beatriz Gómez-Santos, Patricia Aspichueta, Nicole Treichel, Thomas Clavel, Johanna Reißing, Tony Bruns, Matthias Bartneck, Marina S. Mazariegos, Justina Clarinda Wolters, Gonzalo Jorquera, Christian Liedtke, Javier Vaquero, Rafael Bañares, Gulcin Cakan-Akdogan, Matías A. Ávila, Ozlen Konu, Francisco Javier Cubero, Yulia A. Nevzorova

**Affiliations:** a Department of Immunology, Ophthalmology and ENT, Complutense University School of Medicine, Madrid, Spain; b Centre for Biomedical Research, Network on Liver and Digestive Diseases (CIBEREHD), Madrid, Spain; c Gregorio Marañón Health Research Institute (IiSGM), Madrid, Spain; d Department of Molecular Biology and Genetics, Faculty of Science, Bilkent University, Ankara, Turkey; e Spanish National Centre for Cardiovascular Research CNIC, Madrid, Spain; f Inmunotek, S.L. R&D Department, Punto Mobi, 5. Parque Científico Tecnológico, Alcalá de Henares, Spain; g Nobias Technologies, Moscow, Russian Federation; h Department of Microbiome Science, Max Planck Institute for Biology, Tübingen, Germany; i Hepatology Program, CIMA, University of Navarra, Pamplona, Spain; j Upper Gastrointestinal Cancer Translational Research Group, Vall d'Hebron Institute of Oncology (VHIO), Barcelona, Spain; k Gastrointestinal and Endocrine Tumor Unit, Vall d'Hebron Institute of Oncology (VHIO), Hospital Universitari Vall d'Hebron, Vall d'Hebron Barcelona Hospital Campus, Barcelona, Spain; l Izmir Biomedicine and Genome Center, Balcova, Izmir, Türkiye; m Izmir International Biomedicine and Genome Institute, Dokuz Eylül University, Balcova, Izmir, Türkiye; n Hepatic and Intestinal Immunobiology Group, Clinical Medicine Department, Miguel Hernández University, San Juan de Alicante, Alicante, Spain; o IIS ISABIAL, Dr. Balmis University General Hospital, Alicante, Spain; p IDIBE Institute, Miguel Hernández University, Elche, Spain; q Endocrinology Department, Vall d’Hebrón University Hospital, Vall d’Hebron Institute for Research (VHIR), Barcelona, Spain; r Liver Unit, Vall d’Hebron University Hospital, Vall d’Hebron Institute for Research, Barcelona, Spain; s Department of Physiology, Faculty of Medicine and Nursing, University of the Basque Country UPV/EHU, Leioa, Spain; t Biobizkaia Health Research Institute, Barakaldo, Spain; u Functional Microbiome Research Group, Institute of Medical Microbiology, University Hospital of RWTH Aachen, Aachen, Germany; v Department of Internal Medicine III, University Hospital RWTH Aachen, Aachen, Germany; w DWI – Leibniz Institute for Interactive Materials, Aachen, Germany; x Institute of Technical and Macromolecular Chemistry, RWTH Aachen University, Aachen, Germany; y Division of Pediatrics, Department of Clinical Sciences, Lund University, BMC B11, Lund, Sweden; z Department of Pediatrics, University of Groningen, University Medical Center Groningen, Groningen, The Netherlands; aa Institute of Nutrition and Food Technology (INTA), Universidad de Chile, Santiago, Chile; ab Physiology Institute, Science Faculty, Universidad de Valparaíso, Valparaíso, Chile; ac Digestive Service, Gregorio Marañón University General Hospital, Madrid, Spain; ad Department of Medical Biology, Faculty of Medicine, Dokuz Eylül University, Balcova, Izmir, Turkey; ae Healthcare Research Institute of Navarre (IdiSNA), Pamplona, Spain; af Department of Neuroscience, Bilkent University, Ankara, Turkey; ag UNAM-Institute of Materials Science and Nanotechnology, Bilkent University, Ankara, Turkey; ah Institute for Research in Neurochemistry (IUIN), Complutense University of Madrid, Madrid, Spain

**Keywords:** Gut–liver axis, steatohepatitis, CPT1, MASLD, ALD, MetALD, animal model of fibrosis

## Abstract

**Objective:**

In the present study, we investigated the role of the gut–liver crosstalk in the pathogenesis of steatotic liver disease (SLD) induced by the compounding and deleterious effects of alcohol and metabolic risk factors, and explored the potential translational aspects of microbiome-based interventions.

**Design:**

The effects of combined exposure to alcohol and a high-fat, high-cholesterol diet (HFHC) Western diet (WD) were tested in a dietary mouse DUAL model and compared to mice fed only with WD. Liver and gut phenotypes were evaluated via histochemistry, flow cytometry, gene expression, proteomic, and metabolomic analyses. The effects on the gut microbiota were studied in both DUAL mice and MASLD patients with a history of alcohol consumption. Antibiotic-induced microbiota depletion (AIMD) and microbiota modulation therapies (probiotics and fecal microbiota transplant (FMT)) were performed in mice. Primary human hepatocytes and HepG2 cells were used to study the underlying mechanisms. Zebrafish larvae exposed to alcohol and a HFHC diet were used as a validation model.

**Results:**

Alcohol in combination with WD synergistically exacerbated SLD. DUAL-diet-induced disruption of the intestinal barrier led to LPS leakage into the bloodstream and subsequent TLR4-mediated hepatic inflammation. This, together with enhanced intestinal fat absorption, and impaired intrahepatic lipid oxidation – particularly due to insufficient CPT-1 activity – contributed to prominent steatohepatitis. The DUAL-induced changes in the gut microbiota showed similarities to human dysbiosis in MASLD patients who consumed alcohol, including an increase in *Bacteroides* and *Alistipes*. AIMD improved pathology, indicating a causal role of the microbiota in the pathophysiology of DUAL steatohepatitis, whilst early microbiome modulation via FMT induced mild improvements in liver and gut physiology.

**Conclusions:**

These results indicated that the microbiota‒gut‒liver axis plays a crucial role in the progression of SLD intensified by alcohol and concurrent metabolic risk factors, thus providing a promising translational target for potential therapeutic interventions.

## Key messages

**What is already known on this topic:** SLD is a multifactorial, systemic, and multi-axis disease. Increasing evidence points towards a crosstalk between the gut, including the microbiota, and the liver.

**What this study adds:** This study enhances our understanding of SLD of multifactorial etiology induced by metabolic risk factors and simultaneous alcohol consumption. Using the preclinical murine DUAL model and in-depth multi-omics data analysis, we unveiled, for the first time, the importance of the gut‒liver axis for disease progression. We showed that the disruption of gut barriers and increased intestinal permeability affect the balance of inflammatory mediators and lipid metabolism, particularly hepatocytic CPT-1c, and thus promote steatohepatitis. Moreover, we confirmed our data *in vitro* and showed a certain similarity between dysbiosis in DUAL-fed mice and MASLD patients who consumed alcohol. Furthermore, we underscore the potential of microbiome modulation as a novel therapeutic strategy. Finally, we showed that alcohol in combination with metabolic factors causes a profound aggravation of gut and liver damage.

**How this study might affect research, practice or policy:** Our results clearly show that promoting abstinence in addition to reducing the metabolic burden could be a useful and recommended regime for patients with MASLD and MetALD. Moreover, the discovery of unknown mechanisms in the gut–liver axis may open novel therapeutic avenues for the treatment of patients with SLD of “dual” etiology.

## Graphical Abstract

**Figure:**
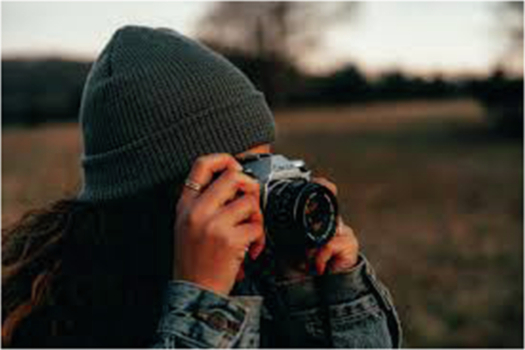

## Introduction

Steatotic liver disease (SLD) has been classified as one of the most prevalent liver diseases worldwide, affecting more than 25% of the global population and representing a considerable public health burden.^1^ SLD serves as an overarching umbrella term comprising various etiologies of steatosis: metabolic dysfunction-associated steatotic liver disease (MASLD), formerly known as non-alcoholic fatty liver disease (NAFLD), as well as alcohol-related liver disease (ALD).^2^

Metabolic syndrome (MS) and alcohol are the leading causes of SLD. However, due to the very high prevalence of both conditions in the same individual, the overlapping of MASLD and ALD is plausible.^3^ Recently, the new term metabolic and alcohol-associated liver disease (MetALD) was selected^2^ to outline a group of patients with MASLD and elevated alcohol consumption (140–350 g/week for females and 210−420 g/week for males).^4^^,^^5^ Furthermore, the ongoing rise in the prevalence of SLD, largely attributed to MASLD, may be influenced by unreported harmful alcohol use.^1^

The deleterious association of alcohol consumption and MS synergistically increases the development of hepatic fibrosis, cirrhosis, and hepatocellular carcinoma (HCC).^6^^,^^7^ Yet the multifactorial pathogenesis of this dangerous combination is poorly understood.^1^

Recent evidence showed that SLD is a systemic disease encompassing white adipose tissue (WAT) failure, hepatic and systemic insulin resistance (IR), dyslipidemia, sarcopenia and cardiovascular diseases.^8^^,^^9^ Moreover, it is well-known that alcohol is primarily absorbed in the stomach and small intestine before reaching the liver and similarly, lipids are also digested in the gut.^10^ Close connection and rigorous mutual collaboration between the gut and the liver form a functional unit called the “gut–liver axis.” Portal circulation is the anatomical link that exists between both organs. Nonetheless, the intestinal barrier limits the extent to which the intestine and liver are connected.^11^^,^^12^ Thus, excessive alcohol^11^ and high-fat high cholesterol diet (HFHC)^13^ could synergistically disrupt the gut–liver axis at multiple interconnected levels leading to gastrointestinal inflammation and dysbiosis, inducing gut damage and increasing intestinal permeability, which altogether increases microbial exposure and generates a proinflammatory environment in the liver.^14^

Identifying the crucial components of gut–liver axis that are most affected in SLD unfolds options for therapeutic intervention.^11^ Nevertheless, the current molecular basis to design such therapeutic trials is weak and has not been studied before for the patients with metabolic dysfunction and simultaneous chronic alcohol consumption.

Recently, we established^15^ a novel preclinical murine experimental model that closely mimics pathogenesis, recapitulates histological, metabolic and transcriptomic gene signature of SLD^3^ and resembles the compound “dual” effects of alcohol and HFHC western diet (WD). At 23 weeks, in addition to obesity, animals develop metabolic syndrome, steatohepatitis, and advanced hepatic fibrosis.^15^^,^^16^

Using the DUAL model, in the present study, we performed an in-depth analysis of the pathomechanisms and functional circuits of hepatic steatosis and inflammation triggered by the synergistic effect of alcohol and metabolic factors and underscored the potential benefits of gut-targeted therapeutic interventions in preventing the progression of SLD of “dual” etiology.

## Methods

### Animal experimentation

#### Mouse feeding

Animals were fed a DUAL diet (Western diet D16022301 (Research Diets, NJ, USA) and 10% alcohol in sweetened drinking water) for a short-term (10 weeks) or long-term (23 weeks) period, as previously described.^15^^,^^16^ DUAL-fed animals were compared to a group fed only a WD and sweetened drinking water and to a control group that received a chow diet and sterilized tap water during the entire treatment period. At the experimental end point, the mice were fasted for 12 h prior to being sacrificed by an overdose of isoflurane inhalation. All animal experiments were performed according to the Spanish laws and regulations on animal protection (PROEX125.1/20; PROEX397.2/21).

### Human feces samples

Fecal samples from human subjects were obtained from Vall d´Hebron University Hospital, Barcelona, Spain, from 2016 to 2019. Clinical characteristics of the patient cohort are outlined in Supplementary Table 1. The research protocol was approved by the Research Ethics Committee for Medicines of Vall d’Hebron Hospital, PR(AG)320/2018 and PR(AG)388/2021, following the *guidance* on *CPMP*/*ICH*/*135/95 and Real Decreto 1090/2015*. All patients provided informed written consent authorizing the storage and research use of their biological samples.

**Table 1.** Changes in the ratios associated with SCD1 activity. Log2 (robust fold-changes) and Student’s *t*-test Wilcoxon adj *p*-values are indicated. Log2 (robust fold-changes) in red indicates an increment of the activity in DUAL mice.

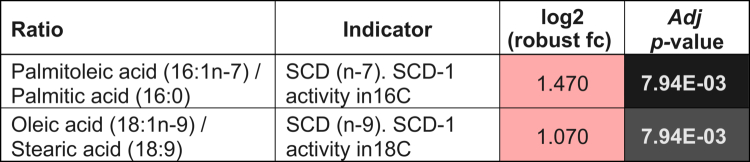

Cases were identified among patients prospectively included in the cohort of steatotic liver disease at the Liver Unit – Chronic Liver Disease Group in Vall d'Hebron University Hospital. The indication for liver biopsy was based on transient elastography and clinical criteria for etiological diagnosis and disease staging. Biopsies were fixed and stained according to the usual standard procedures and analyzed by an experienced pathologist. Control measurements and samples were collected at the Outpatient clinic of the Endocrinology Department of the Vall d´Hebron University Hospital from healthy volunteers recruited via local medical teams from their networks of colleagues and acquaintances with no history of gastrointestinal disease.

### Supplementary materials and methods

Please see the Supplementary Materials, Methods, and Supplementary Tables for additional details on microbiome modulation and characterization, immunofluorescence (IF) and immunohistochemistry (IHC) staining procedures, imaging evaluation, and proteomic and metabolic analysis.

## Results

### Enhanced intestinal fat absorption and altered hepatic fat metabolism in DUAL mice

In our experiments, we fed C57Bl/6Wt male mice a DUAL diet that was composed of WD and 10% alcohol in sweetened (6.75% D-glucose) drinking water for 23 weeks as previously described.^15^^,^^16^ The overlapping features of alcohol and WD leading to SLD were either additive or synergistic: alcohol stimulated the ingestion of WD and vice versa, showing positive feedback resulting in increased daily caloric consumption (Supplementary Figure 1A‒C). Consequently, mice displayed robustly enlarged steatotic livers following 23 weeks of DUAL feeding (Supplementary Figure 2A–F).

**Figure 1.**
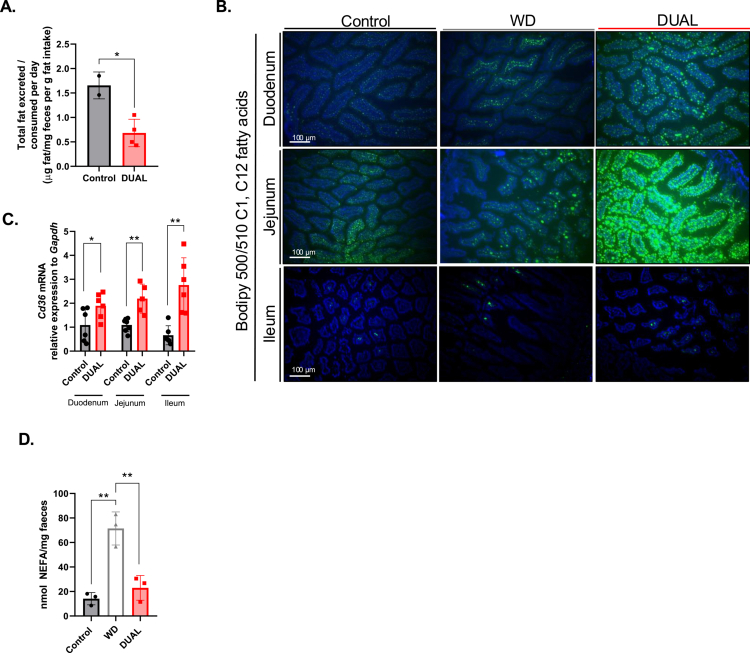
Intestinal fat absorption in DUAL mice. (A) Daily ratio of total fat excreted/consumed (μg fat/mg feces vs. g fat intake/day from diet) in mice (*n* = 2–4). (B) FFA BODIPY-C1, C12 FA in the mouse small intestine. (C) *Cd36* mRNA relative expression to that of *Gapdh* in the mouse small intestine (*n* = 5–6). (D) NEFA quantification in feces (nmol NEFA/mg feces).

**Figure 2.**
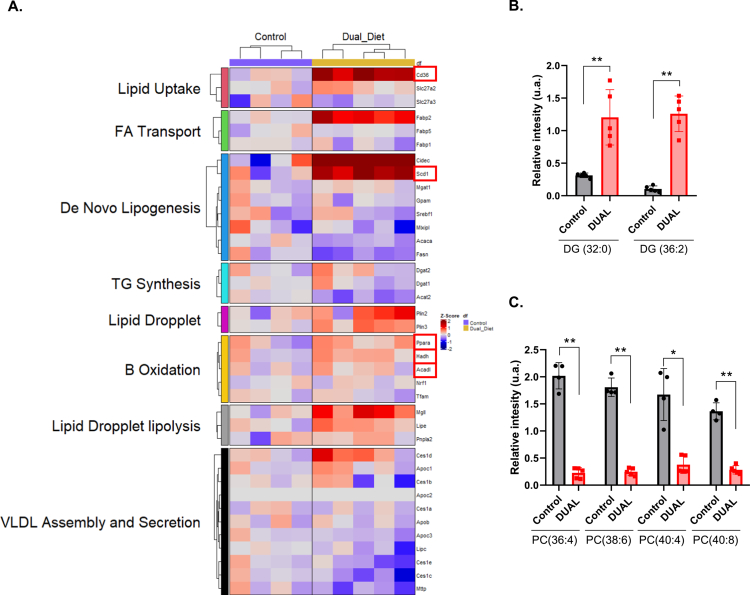
Alcohol in combination with a WD significantly altered hepatic lipid metabolism in mice. (A) Heatmap of the expression of genes grouped into families depending on metabolic process (intake, export, synthesis, or degradation). The expression fold change is compared to that of control animals. (B) Metabolomic study. Plots representing the levels of DG(32:0) and DG(36:2) in liver tissue samples. Data represented as relative intensity (arbitrary units). (C) Metabolomic study. Plots representing the levels of PC(36:4), PC(38:6), PC(40:4), and PC(40:8) in liver tissue samples. Data represented as relative intensity (arbitrary units) (*n* = 5).

The accumulation of fat in the hepatic tissue is caused by an imbalance in metabolism and strongly depends on dietary fat ingestion, absorption, and fecal excretion.^17^^,^^18^ Metabolomic and lipidomic analysis of murine fecal fat showed that most of the metabolites were increased, especially phosphatidyl choline (PC) (Supplementary Figure 3). The low relative ratio of excreted fecal vs. dietary consumed fat was associated with a higher rate of dietary lipid absorption in the intestine (Figure 1A). Indeed, the rate of free fatty acid (FFA) absorption was increased in the duodenum, jejunum, and ileum of DUAL-fed mice (Figure 1B). Similarly, the expression of *Cd36*, a membrane protein that facilitates FFA uptake,^19^ was upregulated in all parts of the small intestine in DUAL animals (Figure 1C). Remarkably, feeding with only a WD for the same period resulted in lower FFA absorption and reduced ileal *Cd36* expression in comparison to DUAL animals (Supplementary Figure 4A). Consistent with these results, fecal non-esterified fatty acids (NEFA) levels were substantially higher in WD-fed mice relative to DUAL group, suggesting a strong positive synergistic effect of WD and alcohol on lipid absorption (Figure 1D).

**Figure 3.**
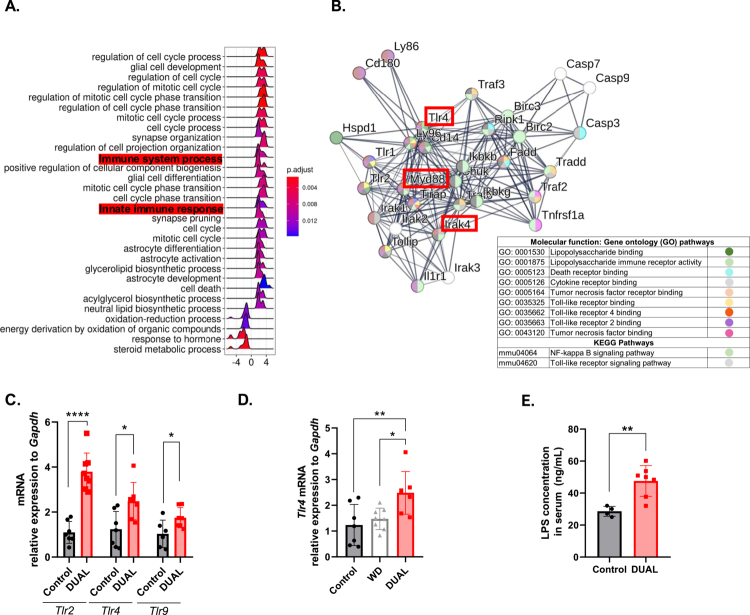
Gut‒liver axis signaling pathways in the liver of DUAL-mice. (A) Analysis of the genes associated with the greatest number of mRNAs. Peaks in negative values represent downregulation, whereas those with positive values represent upregulation. The color scale shows significance from blue to red (*n* = 7). (B) Proteomic analysis of the liver. A network diagram was performed using TLR4 activation found in DUAL livers as a central molecule. GO and KEGG pathways are summarized and classified by colors (*n* = 6). (C) *Tlr2*, *Tlr4,* and *Tlr9* mRNA relative expression to *Gapdh* in the liver. (*n* = 6–7). (D) *Tlr4* mRNA relative expression to *Gapdh* in DUAL vs. WD livers (*n* = 6). (E) LPS in serum (ng/mL) (*n* = 5–7).

**Figure 4.**
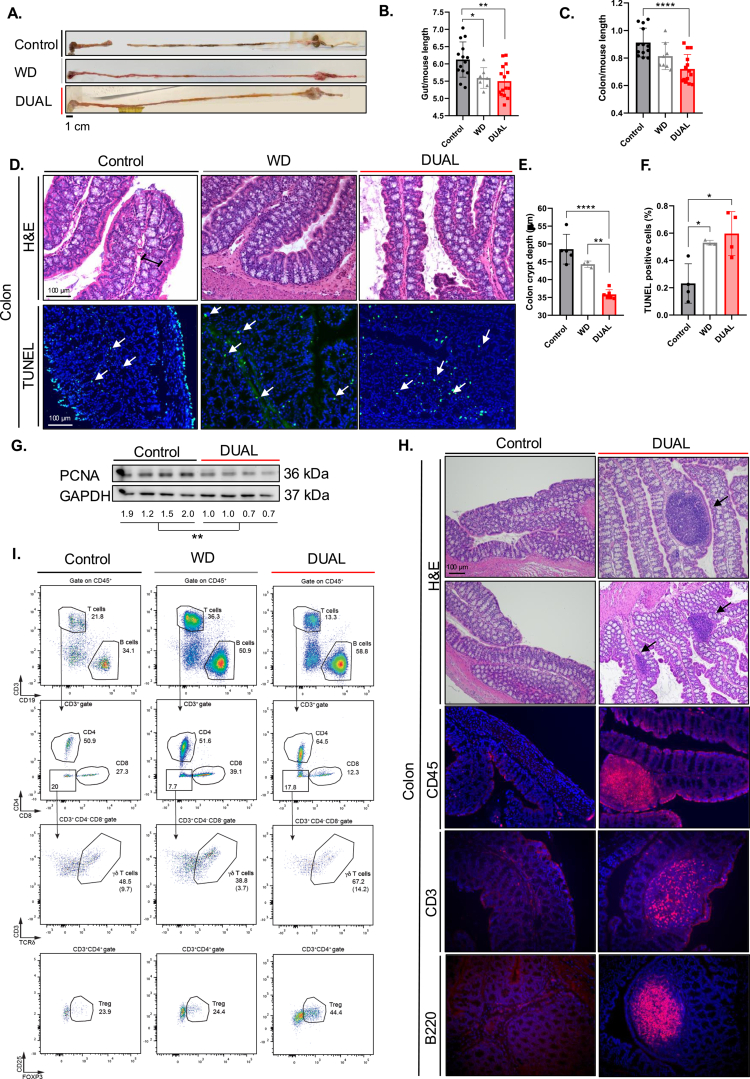
Intestinal alterations induced by DUAL diet. (A) Macroscopic images of the gut. (B) Gut/mouse length ratio (cm/cm) (*n* = 7–14). (C) Colon/mouse length ratio (cm/cm) (*n* = 7–14). (D) Representative H&E images of colon and TUNEL IF, respectively. The arrows point positive stained cells. (E) Colon crypt depth (μm) measurement (*n* = 5–6). (F) Quantification of % TUNEL-positive cells in the colon by ImageJ software (*n* = 3–4). (G) PCNA WB in colon and PCNA/GAPDH protein level ratio (*n* = 4). (H) H&E representative image of GALT in the colon. The black arrow indicates GALT structures. GALT cell type-composition characterization by CD45, CD3, and B220 IF staining in the colon. (I) Flow cytometry dot plots in the colon. The percentages in brackets in the dot plots represent the percentage of the population indicated in the graph (*n* = 4–14).

RNA-bulk sequencing (Figure 2A, Supplementary Figure 5A) and proteomic analysis (Supplementary Figure 5B) of murine livers showed profound alterations of the hepatic lipid metabolism-related factors, such as strong upregulation of the mRNA and protein levels of Cd36, the central regulator of free-fatty acid uptake in DUAL-fed animals (Supplementary Figure 5B, C). Interestingly, the massive FFA influx into the DUAL liver was triggered by the synergistic action of WD and EtOH, as in the mice treated with only a WD mRNA expression of *Cd36* was significantly lower (Supplementary Figure 5C). Moreover, the expression of stearoyl-coenzyme A desaturase 1 (SCD-1), the key regulator of *de novo* lipogenesis, was increased in the hepatic tissue of DUAL mice (Supplementary Figure 5D). SCD-1 is the rate-limiting step in the synthesis of monounsaturated fatty acids (MUFA) – the main components for triglyceride (TG) and diglyceride (DG) production. Similarly, the hepatic levels of MUFA – oleic and palmitoleic acid – were increased, as well as diglyceride (DG) and TG levels in DUAL livers (Table 1; Figure 2B; Supplementary Figure 5E, F).

**Figure 5.**
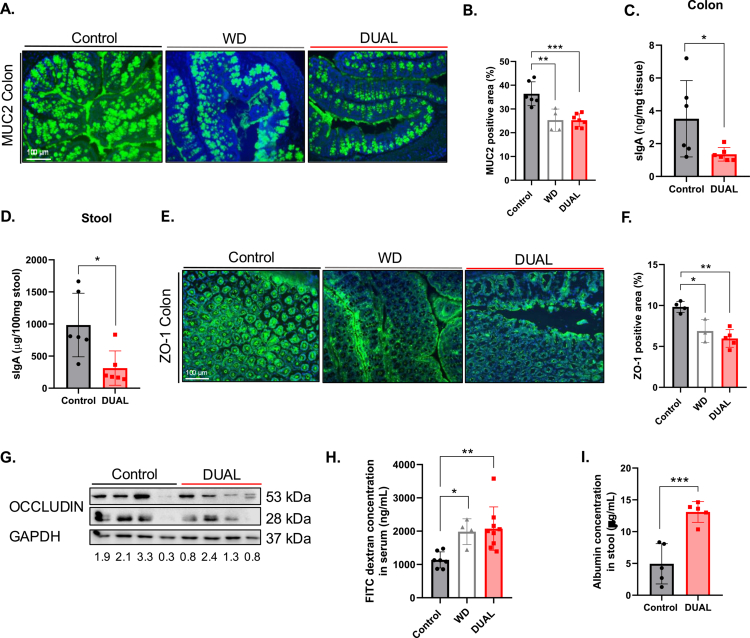
Gut barrier disruption in DUAL-fed mice. (A) IF staining of MUC2 in the colon. (B) Quantification of the positive MUC2-stained area (%) by ImageJ software (*n* = 4–7). (C) sIgA quantification in the colon (ng/mg tissue). (D) sIgA quantification in feces (μg/100 mg stool). (E) IF staining for ZO-1 in the colon. (F) Quantification of the positive ZO-1-stained area (%) by ImageJ software (*n* = 3–5). (G) OCCLUDIN protein level analysis by WB and the OCCLUDIN/GAPDH ratio (*n* = 4). (H) FITC-dextran measurement in serum (ng/mL) (*n* = 4–7). (I) Albumin quantification in feces by ELISA (μg/mL) (*n* = 5).

In contrast, the TG level in the serum of DUAL mice was not proportionally increased and was even lower in comparison to control animals, suggesting that the impairment in the hepatic secretion of very low-density lipoproteins (VLDL) (Supplementary Figure 5G). Indeed, the metabolome study showed a decrease in PC molecules rich in polyunsaturated fatty acids (PUFA) (22:6, 20:4), which are required for VLDL particle formation (Figure 2C). PC(22:6)/PC and PC(20:4)/PE(20:4) ratios related to phosphatidylethanolamine *N*-methyltransferase (PEMT) activity and VLDL assembly were also reduced in DUAL mice (Table 2). RNA-seq confirmed a downregulation of lipid export associated pathways in DUAL mice (Figure 2A).

**Table 2.** Changes in the ratios associated with PEMT activity. Log2 (fold-changes) and Student’s *t*-test *p*-values are indicated. Log2 (fold-changes) in blue denotes reduced activity in DUAL mice.

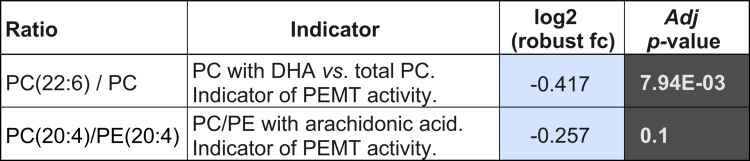

Despite the extraordinary influx of dietary FFA, intensified *de novo* lipogenesis and poor TG excretion, the hepatic lipid *β*-oxidation, carnitine palmitoyltransferase 1a (CPT-1a) (Supplementary Figure 5B) as well as several key regulators of lipid catabolism (e.g., *Ppara, Hadh*, and *Acadl*) (Figure 2A) were not proportionally increased and thus fostered the progression of liver steatosis in DUAL animals.^15^

### DUAL diet-induced hepatitis as a complex interplay linked to lipotoxic species and Toll-like receptor activation

Massive lipid deposition in the liver, remarkable alteration of lipotoxic DG and free cholesterol (FC) together with ingested alcohol contribute to oxidative stress (Supplementary Table 2), liver injury (Supplementary Figure 6), and inflammation.^20^ Flow cytometry analysis of DUAL livers revealed significant increases in monocyte-derived macrophages (MoMFs) (F4/80^+^/Ly6C^+^/CD11b^+^), total T-lymphocytes (CD3^+^) and cytotoxic T lymphocytes (CD8^+^), along with an increase in the population of natural killer (NK) (NK.1.1^+^) cells and a reduction in the population of T helper cells (CD4^+^) (Supplementary Figure 7A–F). Consistently, RNA sequencing confirmed the activation of the innate immune response and the enrichment of gene sets/pathways related to Toll-like receptor (TLR) signaling and NF-κB activation (Figure 3A). The proteomic analysis of DUAL livers showed significant upregulation of TLRs and their corresponding downstream inflammatory-signaling nodes, such as MyD88, NF-κB, and IRAK4 (Figure 3B, Supplementary Table 3).

**Figure 6.**
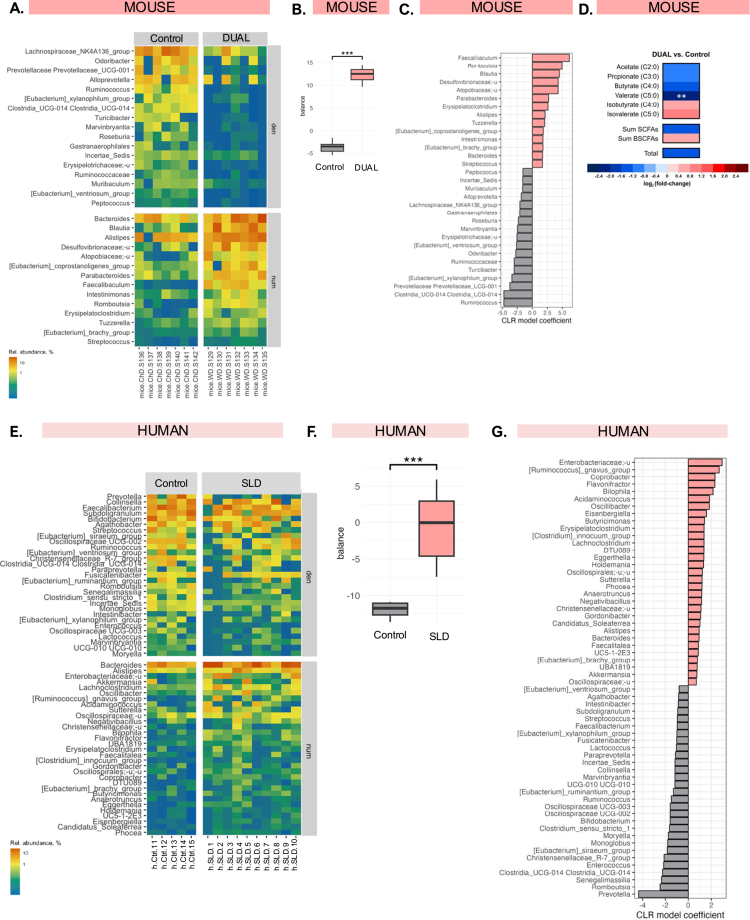
DUAL-fed mice-associated microbiome nearest balance and human SLD-associated microbiome nearest balance. (A) Mouse microbiota member taxa. (B) The normalized ratio of relative abundances between the control and DUAL animal groups. (C) Contribution of each taxon to the balance (calculated as coefficients from a linear model over clr-transformed bacterial abundances) in mice. Bars are filled according to the direction of their association. (D) SCFA heatmap representing the results from the DUAL vs. Control animals unpaired comparison. Heatmap color codes for log2 (pairwise fold-change) are indicated at the bottom of the figure (*n* = 5). (E) Human microbiota member taxa. (F) Distribution of the balance values for each sample compared between the control and SLD patients with alcohol consumption and simultaneous metabolic risk factors. (G) Contribution of each taxon to the balance (calculated as coefficients from a linear model over clr-transformed bacterial abundances) in humans. Bars are filled according to the direction of their association.

**Figure 7.**
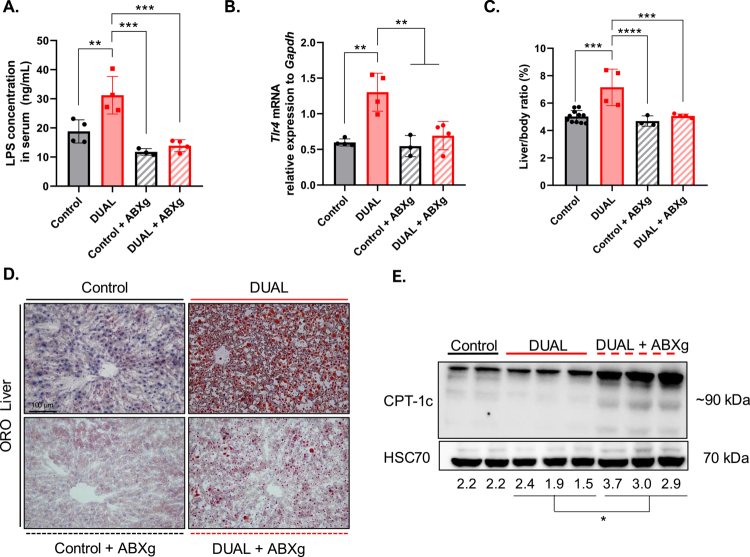
AIMD-associated gut–liver modifications in DUAL mice. (A) LPS in serum by concentrations were measured via ELISA (ng/mL) (*n* = 3–4). (B) *Tlr4* mRNA relative expression to that of *Gapdh* in the liver (*n* = 4–6). (C) Liver/body weight ratio (%) (*n* = 3–5). (D) Illustrative ORO-stained liver sections. (E) CPT-1c WB in the liver; CPT-1c/HSC70 protein level ratio (*n* = 3).

The increased expression of *Tlr2, 4* and *9* was further validated by qPCR analysis (Figure 3C). As the expression of *Tlr4* was significantly lower in mice fed with only WD in comparison to DUAL group (Figure 3D), and we conclude that the combination of alcohol and WD appears to mutually reinforces the activation of TLRs.

TLRs are activated in response to bacterial, viral or fungal subproducts that reach the bloodstream, inducing an immune reaction towards them.^21^ One of the most studied TLR triggers is bacterial surface LPS produced by Gram-negative bacteria in the case of gut barrier leakiness.^22^ Consistently, the serum LPS level is increased in DUAL mice (Figure 3E).

Under normal conditions, LPS or bacterial byproducts are confined to the gut, but can enter the bloodstream when the intestinal barrier is compromised. Thus, we next explored the intestinal changes induced by the DUAL diet.

### DUAL diet induced substantial alteration in gut morphology

DUAL-fed mice showed a significant decrease in overall gut/mouse length, primarily due to a shortened colon (Figure 4A‒C). Histological analysis showed a strong reduction in crypt depth (Figure 4D, E). Gut length reduction and villi/crypt atrophy are recognized as biological markers of intestinal injury.^23^ Accordingly, the pronounced intestinal epithelial cells (IECs) death observed in the colon crypts of DUAL-fed animals (Figure 4D‒F) was not adequately compensated by proliferation (Figure 4G, Supplementary Figure 8A‒C). Notably, alcohol in combination with WD significantly increased the number of morphological changes in the intestine. In contrast, the mice fed only WD did not show significant colon shortening (Figure 4A‒C). Moreover, crypt atrophy in WD-fed animals was mild (Figure 4D,E), and the underlying colonocyte damage (TUNEL staining, Figure 4D–F) was compensated by increased proliferation (*Cyclin A2* expression, Supplementary Figure 8C).

**Figure 8.**
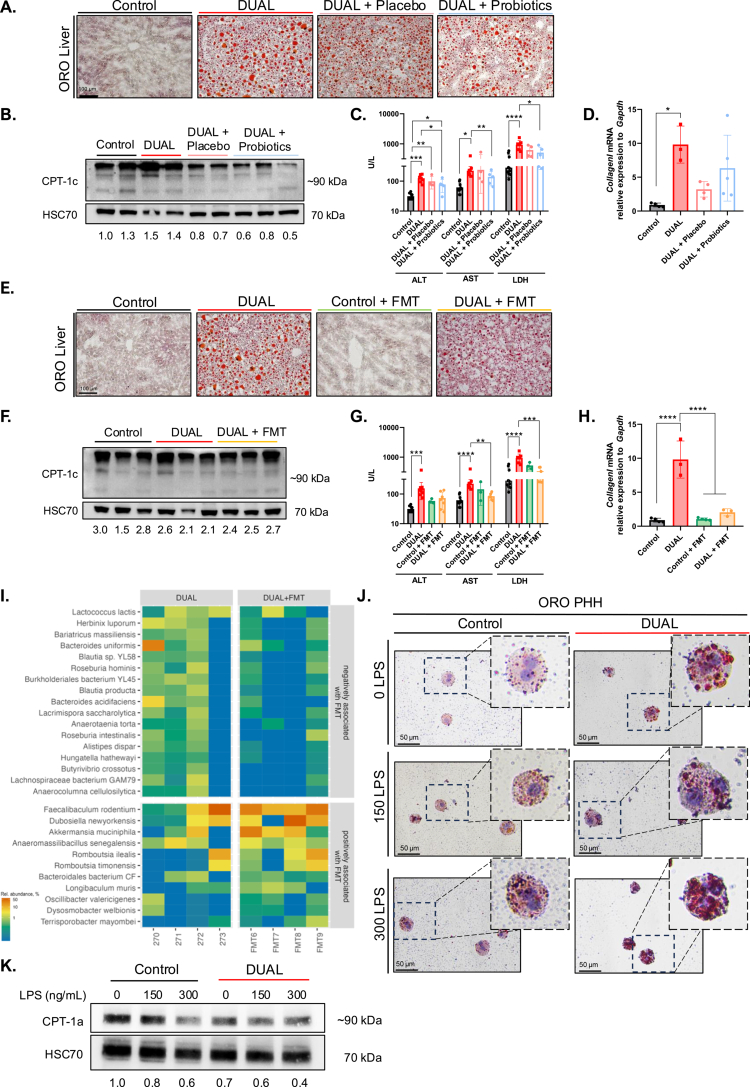
Potential therapeutic role of microbiota modulation in terms of steatosis and crucial role of hepatic CPT-1. (A) Illustrative ORO-staining of the liver after probiotic treatment. (B) CPT-1c WB analysis of the liver with CPT-1c/HSC70 protein level ratio (*n* = 3) after probiotic treatment. (C) ALT, AST, and LDH (U/L) levels in the serum after 12 h of fasting (*n* = 4–5). (D) Hepatic *CollagenI* mRNA relative expression to *Gapdh* (*n* = 4–5). (E) ORO-stained liver sections after FMT treatment. (F) CPT-1c WB analysis of the liver and CPT-1c/HSC70 protein level ratio (*n* = 3) after FMT performance. (G) ALT, AST, and LDH (U/L) levels in the serum after 12 h of fasting (*n* = 4–5). (H) *CollagenI* mRNA relative expression to *Gapdh* in the liver (*n* = 3–4). (I) Relative abundance of the taxa differentiating DUAL and DUAL + FMT according to balance (*n* = 4). (J) ORO-stained PHH after treatment with *in vitro* DUAL diet + LPS. (K) CPT-1a WB and CPT-1a/HSC70 protein level ratios in DUAL *in vitro* + LPS-treated PHH.

Multiple gut-associated lymphoid tissue (GALT) structures were present in the colon of DUAL mice (Figure 4H) and mainly consisted of T-lymphocytes (CD45^+^/CD3^+^) and B-lymphocytes (CD45^+^/B220^+^), with a minor presence of macrophages (CD45^+^/F4/80^+^) and monocytes (CD45^+^/CD11b^+^) (Figure 4H; Supplementary Figure 9A). Subsequently, flow cytometry analysis clearly identified increased populations of T- and B-lymphocytes (Figure 4I, Supplementary Figure 9B, C), but there were no differences in T-helper cells (Figure 4I, Supplementary Figure 9D) and a significant increase in cytotoxic CD3^+^/CD8^+^ cells in the colon of DUAL mice (Figure 4I, Supplementary Figure 9E). Additionally, DUAL feeding led to a reduction in γδ-T-cells, a phenomenon associated with severe intestinal inflammation ^24^ (Figure 4I, Supplementary Figure 9F), and an increase in regulatory T-cells (T-reg) (CD4/FOXP3^+^) (Figure 4I, Supplementary Figure 9G). Finally, the significant upregulation of the proinflammatory cytokine *Tnf-α* was detected in the colons of DUAL-fed animals (Supplementary Figure 9H). In the group of mice fed with only WD, a 2.5-fold increase in the population of B-lymphocytes (CD19^+^/CD45^+^) was identified, while T-lymphocytes and particularly cytotoxic CD8^+^/CD3^+^ cells, were not affected (Figure 4I, Supplementary Figure 9B–E, I), γδ-T-cells remained unchanged and T-reg cells (CD4/FOXP3^+^) significantly increased altogether suggesting a milder intestinal inflammatory response in WD-fed animals than in comparison to DUAL animals (Figure 4I, Supplementary Figure 9F, G).

### The DUAL diet weakened the intestinal barriers and increased gut permeability

In agreement with previous publications,^25^ WD negatively impacts the intestinal barrier by affecting mucin production and tight junctions (TJs) expression, altogether leading to increased intestinal permeability (Figure 5A,B,E,F,H). Similarly, upon DUAL feeding, IF staining of mucin-2 (MUC2) glycoprotein in the colon showed a significant decrease (Figure 5A,B). Loss and damage of the colonic mucus layer induced by DUAL diet were associated with a significant reduction in secretory IgA (sIgA), a key regulator of barrier immune homeostasis (Figure 5C,D).

In addition, a mild decrease in the number of colonic TJs: zona occludens-1 (ZO-1) and occludin, was detected in DUAL-fed animals (Figure 5E‒G).

At the same time, the expression of CD34, a transmembrane glycoprotein of the gut vascular barrier (GVB),^26^ was decreased in the colon (Supplementary Figure 10A, B).

Alterations in all layers of the gut barrier should lead to increased intestinal leakiness.^11^ Hence, a significant increase in FITC dextran in the serum (Figure 5H) and increased albumin concentration in the feces of DUAL-fed animals confirmed disrupted GVB^27^ and intestinal barrier dysfunction (Figure 5I).

### DUAL diet triggered changes in the gut microbiota

Gut microbiome alteration is another key contributor to a dysfunctional gut‒liver axis induced by diet or alcohol.^28^ 16S rRNA gene amplicon sequencing revealed that the fecal microbiota composition was significantly different between control and DUAL mice – at the level of genera and families (PERMANOVA over Euclidean distance for *clr*-transformed data, *p* < 0.001) (Figure 6A). We compared the groups by deriving the nearest balance, which was defined as two subsets of taxa (numerator and denominator, respectively)^29^ The normalized ratio of relative abundances effectively differentiated the groups (Figure 6B). The microbial taxa increased in DUAL animals included those previously linked to high fat and sugar consumption^30^ in humans, such as *Bacteroides, Parabacteroides, and Alistipes,* and unclassified genera within the family Desulfovibrionaceae (Figure 6C).

We also found a decrease in fecal short chain fatty acids (SCFA), specifically valerate, in DUAL mice. This has been previously linked to an increase in gut permeability, gut inflammation, and liver fibrosis (Figure 6D).^31^^,^^32^

Despite a small sample size, the balance analysis along with the ordination plot showed that the gut microbial communities in mice fed with only WD differed from those in the DUAL group, suggesting that the addition of alcohol to the WD led to enhanced disruption of microbial populations (Supplementary Figure 11).

Next, we investigated the potential similarities between the microbiota changes in DUAL mice and obese patients with MASLD and mild, albeit regular, alcohol consumption (Supplementary Table 3). The general difference in fecal microbiota composition between control individuals and SLD patients (genus-level PERMANOVA: *p* = 0.003) was further explored to determine the nearest balance, including 31 genera in the numerator and 29 in the denominator (Figure 6E,F). The topmost taxa associated with the disorder were *Enterobacteriaceae*, *Ruminococcus gnavus*-related taxa, and *Flavonifractor* (Figure 6G). Certain patterns mirrored those observed in DUAL mice, including an increase in *Bacteroides* and *Alistipes*.

### AIMD attenuated hepatic steatosis and inflammation in DUAL-fed animals

To validate whether the phenomenon of DUAL-diet-induced liver damage is mediated by the gut microbiome, we performed antibiotic-induced microbiota depletion (AIMD) by administering a broad-spectrum antibiotic mixture via oral gavage. 16S rRNA gene amplicon sequencing revealed notable depletion of microbial taxa after antibiotic administration (Supplementary Figure 12A).

Serum levels of gut-derived bacterial LPS and subsequent TLR4 expression in the liver significantly decreased in DUAL animals treated with antibiotics (Figure 7A,B). These changes had remarkable consequences for liver pathology: attenuated hepatomegaly (Figure 7C), mitigated steatosis (Figure 7D, Supplementary Figure 12B) and decreased levels of hepatic TGs in DUAL-fed mice (Supplementary Figure 12C). The resolution of steatosis was not a result of diminished lipid influx into the liver, as *Cd36* expression was unaltered after antibiotic treatment (Supplementary Figure 12D). However, AIMD increased CPT-1c hepatic expression levels, highlighting that upregulation of lipid oxidation may be a potential mechanism underlying the observed reduction in hepatic steatosis (Figure 7E).

Decreased steatosis attenuated liver damage (Supplementary Figure 12E), inflammation (Supplementary Figure 12F, G) and decreased hepatic collagen deposition in DUAL-fed mice treated with antibiotics (Supplementary Figure 12H).

To further verify these key data, we used a second more physiological model of AIMD and supplemented the drinking water with an antibiotic cocktail during the final 4 weeks of DUAL feeding. Consistently, DUAL + ABXdw significantly reduced liver weight, attenuated liver steatosis, and decreased the content of hepatic TG (Supplementary Figure 13A–D), decreased liver enzymes in the serum (Supplementary Figure 13E), attenuated inflammation (Supplementary Figure 13F) and the expression of profibrotic *Collagen I* in the liver (Supplementary Figure 13G).

### Probiotic administration did not modify DUAL-induced steatohepatitis

Next, we subsequently investigated whether the microbiome represents a potential target for therapeutic interventions in DUAL mice and tested multi-strain probiotics as a potential therapeutic strategy.

Probiotics administration to DUAL-fed mice neither attenuated hepatomegaly nor hepatic steatosis induced by the DUAL diet (Figure 8A, Supplementary Figure 14A–C). Similarly, following probiotic administration, hepatic lipid uptake, as determined by *Cd36* expression, remained unchanged, as well as the lipid oxidation, as quantified by CPT-1c (Supplementary Figure 14D, Figure 8B). Consequently, probiotic treatment had no effect on liver damage and hepatic collagen deposition in DUAL-treated mice (Figure 8C,D). Finally, *Tlr4* mRNA expression, showed no variance between the groups (Supplementary Figure 14E).

### FMT in DUAL-fed animals caused mild therapeutical effects on steatohepatitis

Next, we evaluated the efficacy of FMT from healthy donors via oral treatment in DUAL-fed mice.

Following FMT administration to DUAL animals, only a slight improvement in hepatomegaly (Supplementary Figure 15A) and a modest reduction in hepatic steatosis, associated with a lower hepatic TG content, were observed (Figure 8E, Supplementary Figure 15B, C). *Cd36* expression remained unaltered in DUAL animals after FMT, and a non-significant increase in lipid oxidation, as indicated by CPT-1c levels, was detected (Supplementary Figure 15D, Figure 8F). Consequently, mild attenuation of liver steatosis was associated with a moderate reduction in hepatic injury and fibrosis in DUAL-fed mice after FMT (Figure 8G,H).

Although the positive changes in the hepatic phenotype were moderate and most of the parameters in the DUAL + FMT group did not reach the control levels, slight improvements in intestinal permeability and gut barriers integrity were demonstrated in the colon (Supplementary Figure 15E, F). However, the expression of *Tlr-4* in the liver and LPS levels in the serum remained unchanged after FMT in DUAL animals (Supplementary Figure 15G, H).

No one-directional dynamic in the composition of the intestinal microbiota in DUAL + FMT mice was observed (Supplementary Figure 15I). However, a substantial increase in three commensal bacterial species was observed: *Faecalibacterium rodentium, Dubosiella newyorkensis,* and *Akkermansia muciniphila* (Figure 8I). These species, together with *Dysosmobacter welbionis*, which was also increased in DUAL + FMT mice, albeit at lower relative abundances, are associated with beneficial effects on host metabolism.^33^^,^^34^

### LPS had a direct impact on hepatic lipid metabolism in vitro

To further validate our key findings, we applied an *in vitro* DUAL model to HepG2 human hepatoma cells. First, in HepG2 cells, we examined the effects of palmitic acid (PA) alone and compared it to the combination of PA plus EtOH. Our results supported the *in vivo* findings and clearly demonstrated that the “dual” treatment (PA + EtOH) synergistically increases the lipid accumulation in HepG2 cells (Supplementary Figure 16 A, B).

In our murine DUAL model, the presence of LPS in the serum was associated with the accumulation of fat in the liver due to inadequate CPT-1c-mediated lipid oxidation. Consistently, HepG2 cells treated with PA plus alcohol and the highest concentration of LPS, presented the greatest level of lipid accumulation (Supplementary Figure 16C, D) directly correlated with a reduction in CPT-1a-mediated lipid oxidation (Supplementary Figure 16E).

To further confirm this important finding, we tested the consequences of *in vitro* DUAL treatment (palmitic acid (PA) plus alcohol) in combination with LPS in primary human hepatocytes (PHH). As expected, *in vitro* DUAL treatment of PHHs led to the intracellular accumulation of lipids, which was greatly enhanced after LPS application in a dose-dependent manner (Figure 8J). Remarkably, DUAL + LPS-induced lipid accumulation in PHH was associated with an impairment in CPT-1a protein expression (Figure 8K).

### The combined exposure to alcohol and a HFHC induced hepatic steatosis and intestinal damage in zebrafish larvae

Finally, in order to validate and compare the clinical translatability of DUAL mice, we used a second model organism – zebrafish (*Danio rerio*) larvae.

The mouse phenotype was effectively replicated in zebrafish larvae at 9 days post fertilization (dpf), where the exposure to EtOH and a high-fat, high-cholesterol (*EtOH/*HFHC) diet of egg yolk led to increased hepatic lipid accumulation (Supplementary Figure 17A, B). Histopathological analysis of Alcian blue/PAS staining in zebrafish livers revealed that hepatocytes exposed to EtOH and HFHC diets displayed deformations, ballooning and fat accumulation, resembling the disruption of the hepatic parenchyma, as it occurs in mammals (Supplementary Figure 17C).

Consistently, the Alcian Blue/PAS staining of the intestine of zebrafish larvae simultaneously exposed to EtOH and a HFHC diet showed loosening of the tissue structure, flattening of the crypts and villous atrophy, which is assumed to be a result of the disbalance between IECs proliferation and death (Supplementary Figure 17C).^35^

## Discussion

The “gut–liver axis” refers to the reciprocal and functional interaction between the gut, microbiota, and the liver. Since the gut and liver are interdependent organs, alterations in the intestinal barrier might lead to an increased portal influx of bacteria or their products into the liver, thus triggering a variety of hepatic disorders, including MASLD and ALD.^12^ The combination of MASLD and simultaneous alcohol consumption refers to a distinctive systemic and potentially multiaxial entity, whose pathogenesis remains elusive.^5^^,^^11^ In the present work, we used the preclinical murine DUAL model^15^ to examine the relevance of the microbiome‒gut‒liver crosstalk and explored potential therapeutic circuits.

DUAL feeding induced significant intestinal morphological alterations and augmented IECs death not balanced by compensatory proliferation. This resulted in villi atrophy, epithelial cell gap formation, and permeability changes, exacerbating epithelial shedding and inflammatory responses.^36^^,^^37^

Multiple GALT structures identified in DUAL colon were characterized by expanded populations of T-and B-cells and elevated proinflammatory cytokine levels, that have been reported as main inducers of local immune responses.^38^ Moreover, DUAL feeding led to a significant increase in cytotoxic T cells and a decrease in protective γδ-T-cells, previously linked to severe intestinal inflammation in preclinical studies.^24^ The corresponding low level of sIgA worsened intestinal epithelium protection from pathogenic microorganisms and further disrupted microbiota homeostasis.^39^ Gut damage and inflammation in DUAL mice caused a significant disruption of the mucus layer and TJs, which in combination with loss of the GVB integrity,^40^ increased bidirectional intestinal permeability.^41^

16S rRNA amplicon sequencing of stool samples unveiled microbial dysbiosis in DUAL mice and important shifts in the composition of dominant bacterial families and single species.^42^ Intestinal dysbiosis is a hallmark in multiple diseases, including MASLD and ALD, underscoring its importance in maintaining intestinal health and resilience.^43^^,^^44^ More specifically, a notable increase in *Bacteroides* was identified in DUAL animals, that correlated with fecal content rich in fat and sugars and is known for positive association with MASLD pathogenesis.^45^ This was accompanied by a decrease in *Prevotella* consistent with the competitive relationship between these two genera.^45^ As *Prevotella* synthesize SCFA, in particular valerate, their depletion may have been associated with the reduction in SCFA observed in DUAL mice.^46^^,^^47^ This may have contributed to aggravated loss of intestinal barrier integrity, mucus production, and protection against inflammation.^32^ Importantly, some of the changes in bacterial composition after DUAL diet resembled the patterns of dysbiosis in SLD patients, supporting the relevance of our model.

Diet-induced alterations in microbiota, together with hyperpermeable intestinal barriers facilitated portal influx of LPS to the liver, where it (i) triggered a proinflammatory cascade *via* activation of TLRs corresponding downstream inflammatory-signaling nodes; and (ii) promoted hepatic steatosis via inhibition of ß-oxidation.

Indeed, DUAL animals exhibited an enhanced ability to absorb fat in the intestine. Thus, following 23 weeks of DUAL feeding, we observed marked FFA influx into the liver, combined with intensified *de novo* lipogenesis and impaired export and ß-oxidation of lipids, which, altogether, contributed to progressive steatosis. Our *in vivo* and *in vitro* findings confirm and extend previous studies demonstrating the suppressive effect of LPS on FFA oxidation and, particularly, on its key regulator CPT1. LPS-associated decreased ß-oxidation in hepatocytes could be a by-product of the systemic coordinated host response to increase FFAs and TGs in order to provide lipid substrates for cells that play a crucial role in host defense or tissue repair.^48^^,^^49^

However, the inability of DUAL hepatocytes to degrade FFA by adequate ß-oxidation contributes to hepatic steatosis, triggering oxidative stress, cell death and immune cell infiltration into the liver tissue. In the liver tissue of DUAL-fed mice, we observed an increase in NK cells, MoMFs, cytotoxic T lymphocytes, and a decrease in T helper cells altogether contributing to liver inflammation, injury, and fibrosis.^50^

Indeed, the liver's innate immune response is crucial in the pathogenesis of SLD. Immune cells in the liver recognize cell damage or pathogen invasion, subsequently initiating signaling cascades and further promoting the inflammatory response, resulting in a state of unresolved chronic inflammation.^51^

The dysregulation of T cell populations, with increased CD3/CD8^+^ T lymphocytes and reduced CD4^+^ T cells, mirrored human-like steatohepatitis pathogenesis and correlated with disease progression to more advanced fibrosis.^50^

Importantly, the depletion of bacteria by AIMD significantly reduced LPS serum levels, ameliorated lipid accumulation by increasing the levels of lipid oxidation through CPT-1c, disabled the TLR4 inflammatory cascade and, consequently, the progression of hepatic fibrosis.

Our findings suggest that targeting the imbalanced microbiota in DUAL animals largely prevents SLD development. Nevertheless, it is important to recognize that bacteria symbiotically interact with the host, including metabolism regulation, maintenance of immune homeostasis, and preservation of a healthy gut barrier.^52^

The use of microbiome modulation is a novel therapeutic strategy for SLD. While nutritional and lifestyle interventions remain the primary recommendations for ALD and MASLD, adherence to dietary changes is often challenging.^53^ Thus, in this study, we modified the microbiota in DUAL mice while maintaining their diet. Initially, we explored the therapeutic potential of probiotics supplementation, a method previously suggested to alleviate liver steatosis and inflammation.^54^ However, our findings did not demonstrate significant clinical benefits. We speculate that certain beneficial bacteria within probiotics may struggle to colonize the DUAL gut effectively, potentially owing to the reduced levels of commensal bacteria.^55^

Recognizing the limitations of probiotics supplementation, we proposed an alternative approach: FMT from healthy donors. Animal studies and clinical trials have shown promising results of FMT in improving liver health, gut permeability, and metabolic syndrome.^56^^,^^57^ Our study revealed partial improvement in liver markers of injury after as well as enhancement of the gut phenotype after FMT in DUAL mice. 16S rRNA gene amplicon sequencing showed a tendency toward compositional changes induced by FMT at the species level; a larger sample size would be required to evaluate the specific direction of the shift differentiating these from the DUAL animals and the controls. An increase in some bacterial commensal species, such as *Faecalibacterium rodentium,Dubosiella newyorkensis*, and *Dysosmobacter welbionis* was noted, which have been found to have hepatoprotective properties^58^^,^^59^ and beneficial influence on host metabolism.^32^ Moreover, we observed alterations in other bacterial taxa, such as *Akkermansia muciniphila*, known for its role in maintaining mucosal barrier integrity and overall gut homeostasis.^60^ Our results support the potential of FMT as a therapeutic intervention for SLD, even though its efficacy requires further investigation.

Several important findings in our study represent major novelties over prior work in the field. First, from the current literature, we know that rodents are not the easiest translational model for studying alcohol consumption, as they metabolize alcohol quickly and have a high basal metabolic rate in comparison to humans.^61^ The resemblance between zebrafish larvae and mice regarding the key alterations in the gut and liver not only supports our findings but also paves the way for utilizing efficient, rapid, and cost-effective models to explore the broader aspects of SLD pathophysiology.

Second, the concurrent combination of alcohol consumption with metabolic risk factors should not be considered ALD or MASLD alone. In line with previous observations,^62^ our study provides some insights into the reciprocal interaction between high-fat food and alcohol intake, indicating the possibility that each nutrient stimulates the intake of the other. In DUAL mice, alcohol stimulated the increased consumption of WD and vice versa. This is relevant since such vicious synergism in the DUAL model leads to a significant excess of calories in comparison to WD alone. However, as summarized in Supplemental Figure 18A, the variations observed between DUAL and WD are not only attributable to increased caloric intake. Moreover, DUAL mice have a relatively high rate of intestinal absorption of dietary lipids, leading to the massive FFA flux to the liver. *In vivo* and *in vitro* data confirmed the inability of hepatocytes to degrade lipids by adequate ß-oxidation in response to “dual” damage (EtOH in combination with high fat), which further aggravated hepatic steatosis. Moreover, in the gut, the combination of alcohol and WD has more severe negative consequences for IEC proliferation, leads to more prominent intestinal damage, inflammation and more pronounced microbiome disbalance, which together superactivates the TLR4 inflammatory cascade in the liver and reinforces the progression of hepatic inflammation/fibrosis.

Indeed, the liaison between MASLD and alcohol intake has been a matter of debate in recent years. Our findings highlight the need of encouraging abstinence in addition to lowering the metabolic burden as a beneficial and recommendable therapy for MASLD and MetALD patients. We propose that alcohol-related public health guidelines should incorporate additional public health initiatives addressing the link between alcohol, obesity, and SLD.

In summary, the use of the murine DUAL model, zebrafish larvae, cell lines, and patient cohorts provided evidence suggesting that gut–liver axis disruption plays a pivotal role in the pathogenesis of SLD induced by alcohol and concurrent metabolic risk factors. Gut dysbiosis is a determinant for the development of this dysfunction, and therefore, gut-based early therapeutic interventions^63^ could be beneficial for halting the development of MetALD.

## Supplementary Material

Supplementary material Suppl_figures_with_captions_clean.

## Data Availability

The authors confirm that the data supporting the findings of this study are available within the article.

## Abbreviations

AIMD: Antibiotic-induced microbiota-depletion
ALD: Alcohol-related liver disease
ALT: Alanine aminotransferase
AST: Aspartate aminotransferase
CMF-PBS: Ca^2+^ and Mg^+^-free Phosphate buffered saline, PBS
CPT-1c: Carnitine Palmitoyltransferase-1c
DG: Diglyceride
FA: Fatty acid
FBS: Fetal bovine serum
FC: Free cholesterol
FFA: Free-fatty acid
FMT: Fecal microbiota transplant
GALT: Gut-associated lymphoid tissue
GVB: Gut vascular barrier
HADH: Hydroxyacyl-CoA dehydrogenase
HFHC: High-fat and high-cholesterol
HFD: High fat diet
HCC: Hepatocellular carcinoma
IBD: Inflammatory bowel disease
IEC: Intestinal epithelial cell
IF: Immunofluorescence
IHC: Immunohistochemistry
IR: Insulin resistance
KC: Kupffer cells
LDH: Lactate dehydrogenase
LPS: Lipopolysaccharide
MASLD: Metabolic dysfunction-associated steatotic liver disease
MetALD: Metabolic- and alcohol-associated liver disease
MoMFs: Monocyte-derived macrophages
MS: Metabolic syndrome
MUC2: Mucin-2
MUFA: Monounsaturated fatty acids
MyD88: Myeloid differentiation primary response 88
NAFLD: Non-alcoholic fatty liver disease
NEFA: Non-esterified fatty acid
NF-ĸB: Nuclear factor-kappa B
NK: Natural killer
ORO: Oil red O
OTUs: Operational taxonomic units
PA: Palmitic acid
PC: Phosphatidyl choline
PCA: Principal components analysis
PEMT: Phosphatidylethanolamine N-methyltransferase
PHH: Primary human hepatocytes
PPARα: Peroxisome proliferator-activated receptor alpha
PUFA: Polyunsaturated fatty acids
SCD-1: Stearoyl-coenzyme A desaturase 1
SCFA: Short chain fatty acid
sIgA: Secretory immunoglobulin A
SLD: Steatotic liver disease
TG: Triglyceride
TJ: Tight junction
TLR: Toll-like receptor
T-reg: Regulatory T-cells
TUNEL: Terminal deoxynucleotidyl transferase dUTP Nick end labelling
VLDL: Very low-density lipoproteins
WAT: White adipose tissue
WB: Western blot
WD: Western diet
ZO-1: Zona occludens-1
