# Supplementary material for: Alcohol consumption in metabolic dysfunction-associated steatotic liver disease (MASLD): understanding the gut–liver crosstalk for clinical translation
Source: Gut Microbes. 2026 Feb 21;18(1):2631834. doi: 10.1080/19490976.2026.2631834 (PMC12928667; doi:10.1080/19490976.2026.2631834)
Supplement: Supplementary material — Suppl_figures_with_captions_clean. [file KGMI_A_2631834_SM2906.pdf]

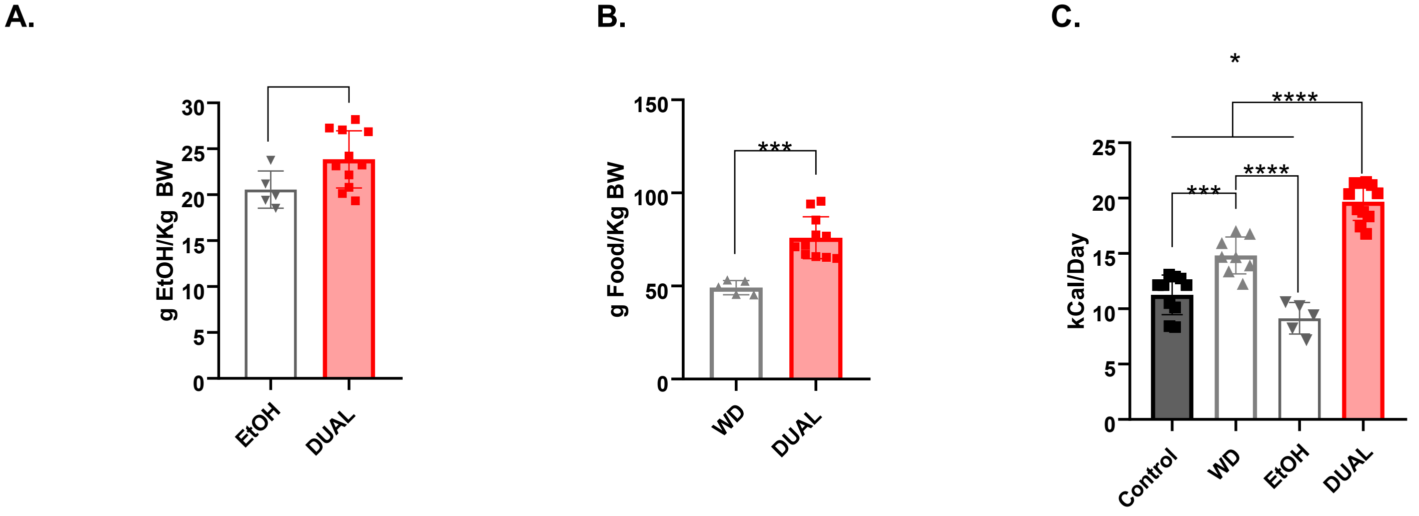

Suppl. Fig. 1. A. Daily EtOH intake (g). Grams of consumed EtOH were calculated according to the body weight (n=5-11). **B.** Daily food intake (g) WD/kg mouse (n=6-11). **C.** Calorie intake per day including calories in food and in drinking water glucose/EtOH (n=8-11).

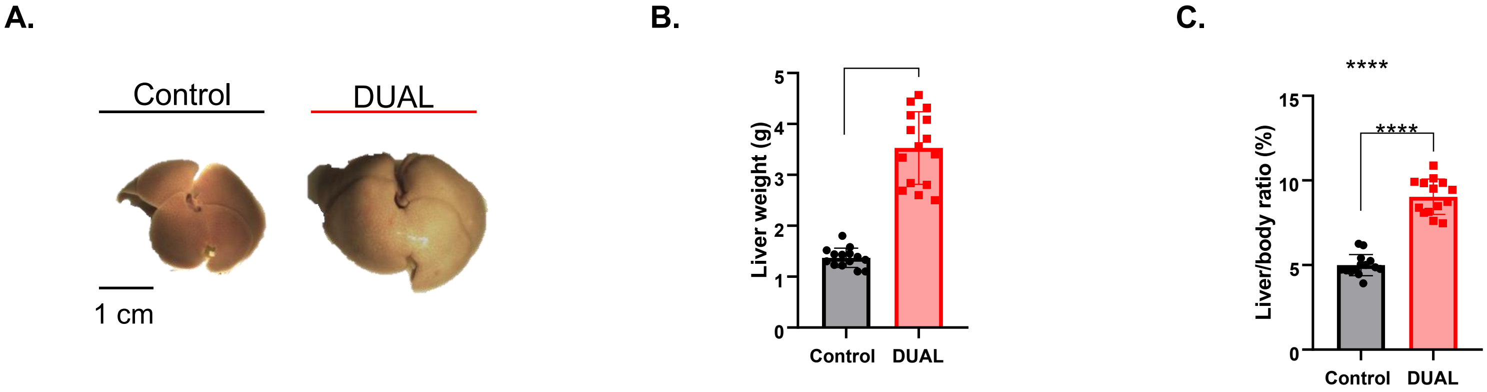

D.

E.

F.

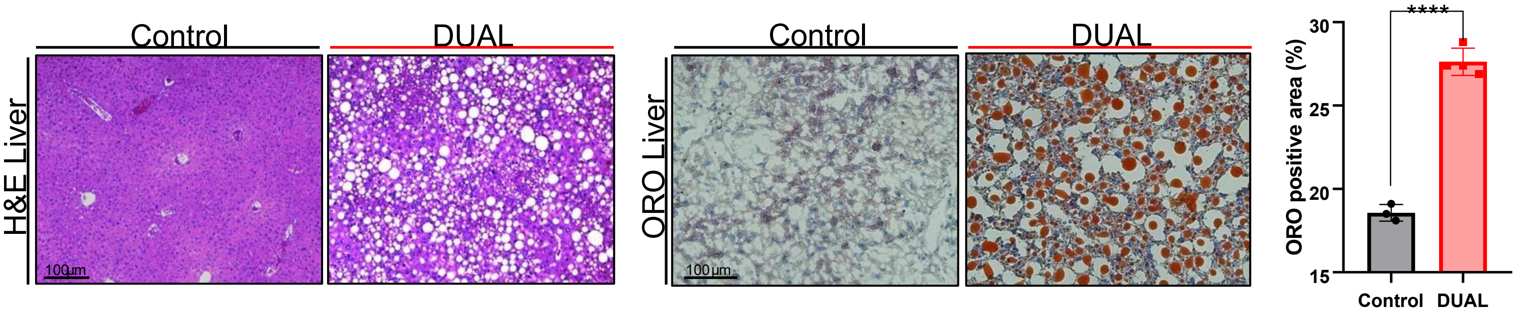

Suppl. Fig. 2. Diet-induced hepatomegaly and hepatic steatosis in DUAL mice. A. Liver macroscopic images of murine liver after 23 weeks of DUAL feeding. **B.** Liver weight (g) (n=14-15). **C.** Liver/body weight ratio (%) (n=14-15). **D.** Hematoxylin and eosin (H&E) representative images of liver in mice after 23 weeks of DUAL feeding. **E.** Illustrative ORO-stained liver sections. **F.** Quantification of ORO-stained area (%) (n=3-4).

A.

B.

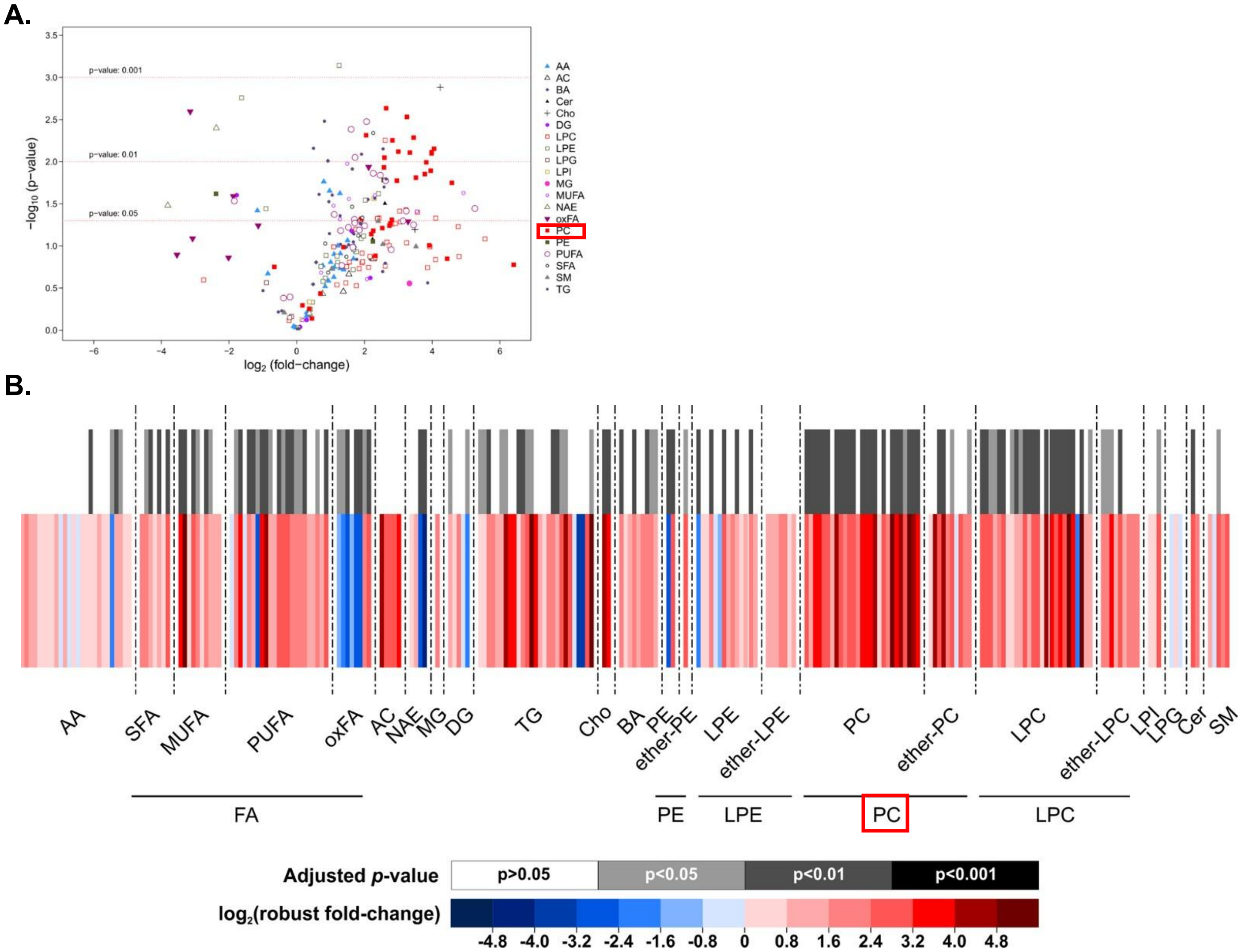

Suppl. Fig. 3. Metabolomic study in feces. **A.** Volcano plot [-log10(p-value) vs. log2(foldchange)] for the comparison DUAL vs. Control mice (fecal samples). Abbreviations: AA, amino acids; AC, acylcarnitines; BA, bile acids, Cer, ceramides; Cho, cholesterol; DG, diglycerides; LPC, lysophosphatidylcholines; LPE, lysophosphatidylethanolamines; LPG, lysophosphatidylglycerols; LPI, lysophosphatidylinositols; MG, monoglycerides; MUFA, monounsaturated fatty acids; NAE, Nacyl ethanolamines; oxFA, oxidized fatty acids; PC, phosphatidylcholines; PE, phosphatidylethanolamines; PI, phosphatidylinositols; PUFA, polyunsaturated fatty acids; SFA, saturated fatty acids; SM, sphingomyelins; TG, triglycerides. **B.** Heatmap representing binary comparisons between DUAL and Control per metabolite (Study of fecal samples). Heatmap color codes for log2 (fold-change) and Student's t-test adjusted p-values (Wilcoxon post-hoc) are indicated at the bottom of the figure (n=5).

A.

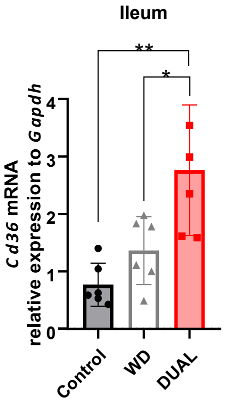

Suppl. Fig. 4. Intestinal fat absorption DUAL vs. WD mice. A. *Cd36* mRNA relative expression to *Gapdh* in mouse small intestine (n=5-6).

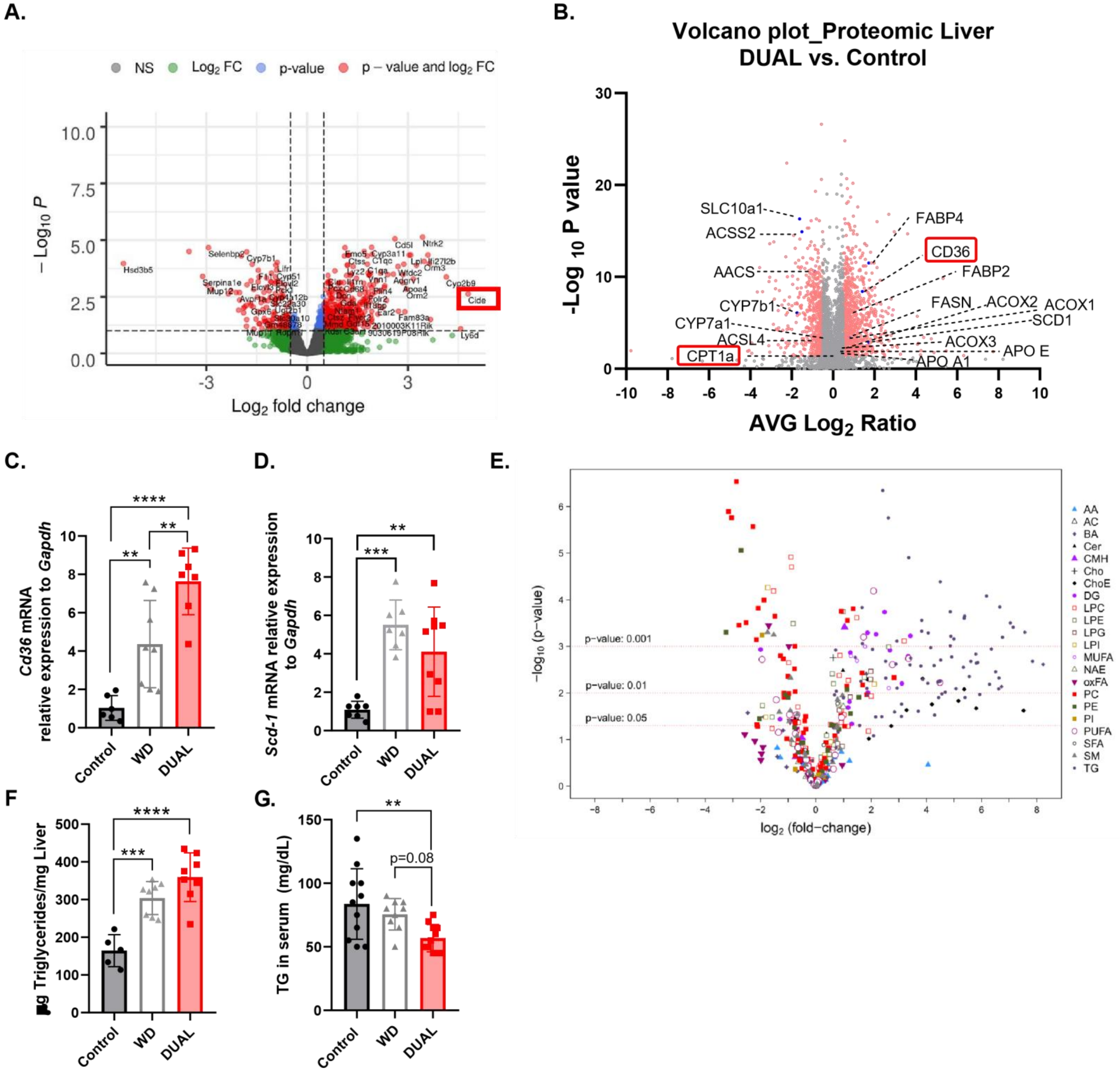

Suppl. Fig. 5. Changes in fat metabolism associated with DUAL-feeding in mice. A. Volcano plot 3'mRNA sequencing analysis comparing DUAL to control animals. **B.** Volcano plot proteomic study in the liver comparing DUAL to control animals. Lipid metabolism related genes are highlighted. **C-D.** *Cd36 and Scd-1* mRNA relative expression to *Gapdh* was determined respectively in liver tissue by RT-qPCR. (n=5-8). **E.** Metabolomic study in the liver. Volcano plot [-log10(p-value) vs. log2(foldchange)] for the comparison DUAL vs. Control mice (hepatic tissue samples). Abbreviations: AA, amino acids; AC, acyl carnitines; BA, bile acids, Cer, ceramides; CMH, monohexosylceramides; Cho, cholesterol; ChoE, colesterol esters; DG, diglycerides; LPC, lysophosphatidylcholines; LPE, lysophosphatidylethanolamines; LPG, lysophosphatidylglycerols; LPI, lysophosphatidylinositols; MG, monoglycerides; MUFA, monounsaturated fatty acids; NAE, N-acyl ethanolamines; oxFA, oxidized fatty acids; PC, phosphatidylcholines; PE, phosphatidylethanolamines; PI, phosphatidylinositols; PUFA, polyunsaturated fatty acids; SFA, saturated fatty acids; SM, sphingomyelins; TG, triglycerides. **F.** Quantification of hepatic TG (µg/mg liver) (n=5-8). **G.** TG in serum (mg/dL) after 12 h fasting (n=9-11).

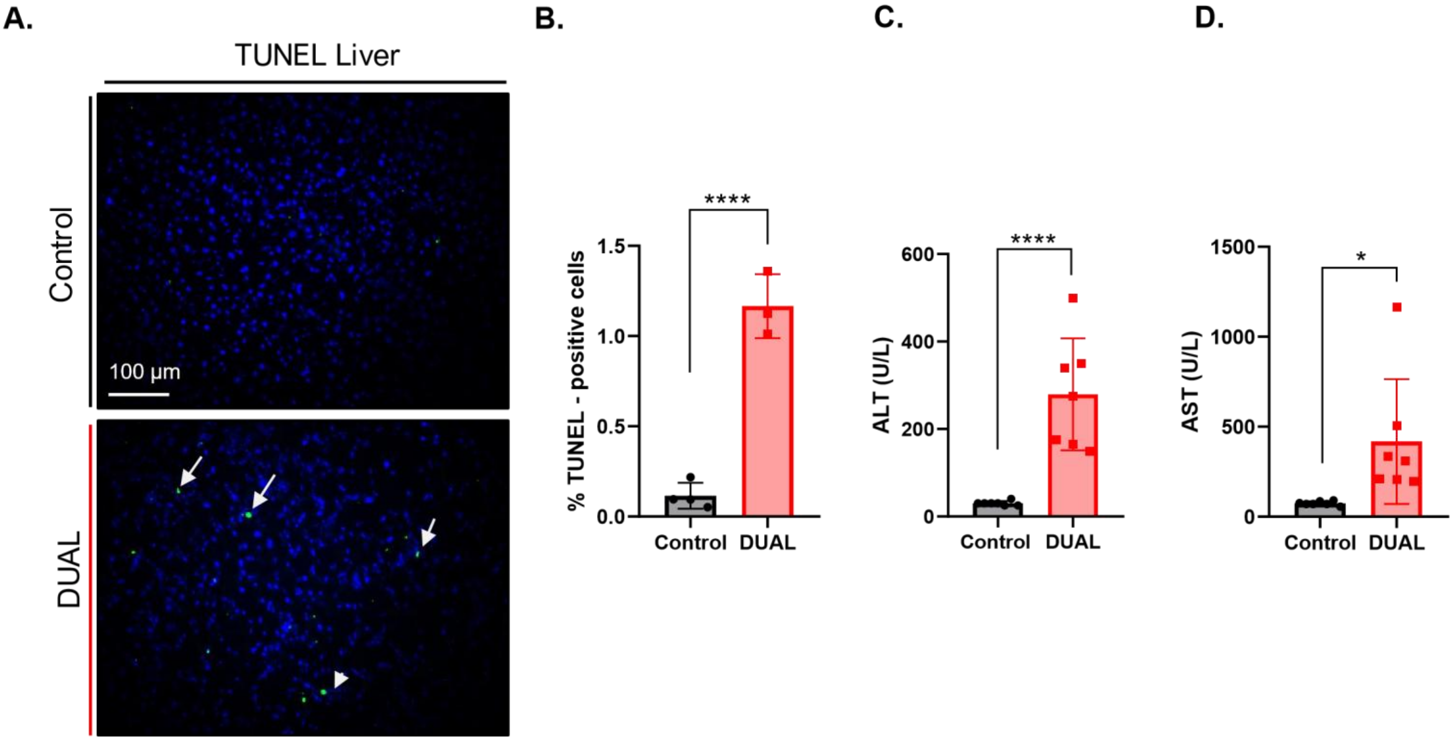

Suppl. Fig. 6. Cell death and liver damage induced by DUAL diet. A. TUNEL IF staining in colon. TUNEL+ cells are labeled in green. DAPI (blue) was used as counterstain. Arrows point to TUNEL+ cells. Scale = 100 µm. (n=3-4). **B.** Quantification of %-TUNEL positive cells in liver by Image J software (n=3-4). C-D. ALT and AST (U/L) in serum respectively. (n=7).

A.

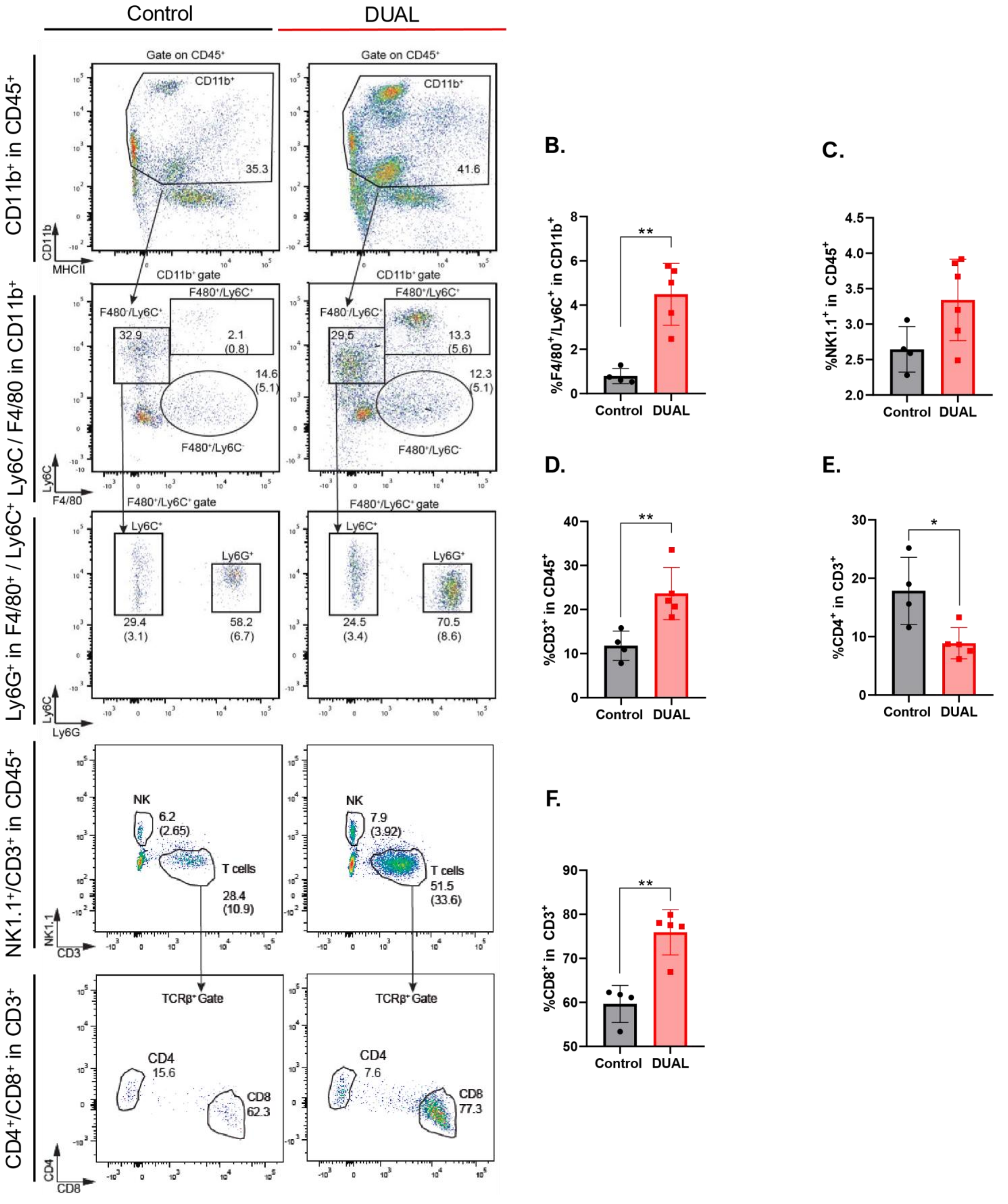

Suppl. Fig. 7. DUAL-induced hepatitis in mouse liver. A. Flow cytometry dot plots in liver. Percentages in brackets in the dot plots represent the % of the population indicated in the graph. B. %F4/80$^+$/ Ly6C$^+$ in CD45$^+$/CD11b$^+$. **C. %**NK1.1 in CD45$^+$. D. %CD3 in CD45$^+$. **E.** %CD4 in CD3$^+$. **F.** %CD8 in CD3$^+$. (n=4-5).

A. **B.** **C.**

Ki-67 Colon

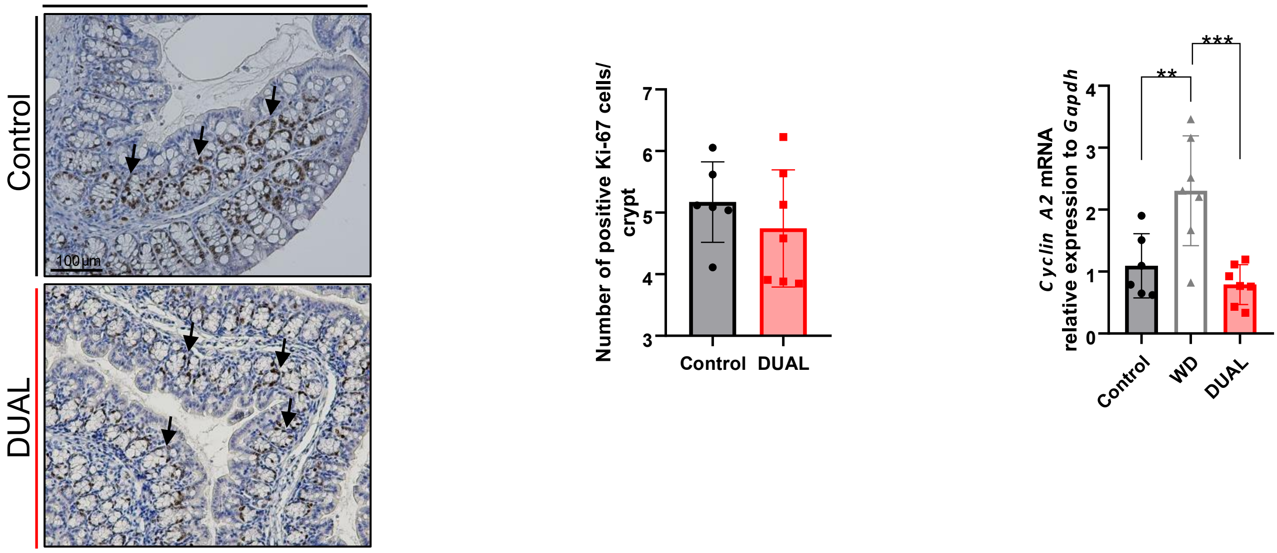

Suppl. Fig. 8. Phenotypic changes in colon. A. Ki-67 IHC in colon. Arrows point positive stained cells. **B.** Number of positive Ki-67 cells in 20x area (n=6). **C.** *Cyclin A2* mRNA relative expression to *Gapdh* in colon (n=6-7).

A.

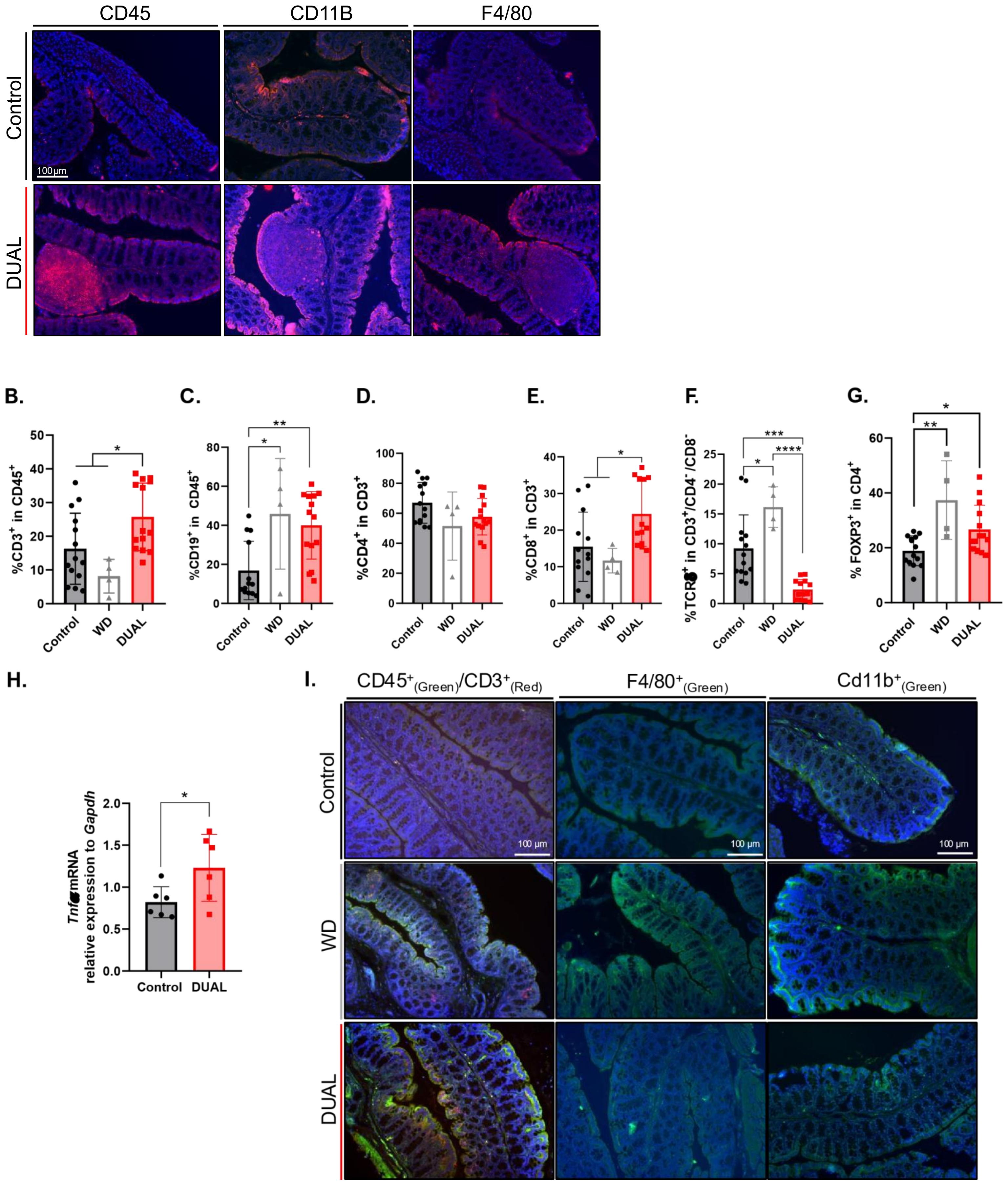

Suppl. Fig. 9.

Suppl. Fig. 9. Diet induced inflammation in colon of DUAL mice. A. GALT cell type-composition characterization by CD45, CD11B, F4/80 IF staining. **B.** %CD3 in CD45+ analyzed by flow cytometry in colon. **C.** %CD19 in CD45+ analyzed by flow cytometry in colon. **D.** %CD4 in CD3+ analyzed by flow cytometry in colon. **E.** %CD8 in CD3+ analyzed by flow cytometry in colon. **F.** %TCRδ+ in CD3+

analyzed by flow cytometry in colon. **G.** %FOXP3 in CD4+ analyzed by flow cytometry in colon. **H.** *Tnf-α* mRNA relative expression to *Gapdh* in colon (n=7). **I.** $CD45^+/CD3^+$, $F4/80^+$ and $Cd11b^+$ IF in colon. $CD3^+$ cells are shown in red. $CD45^+$, $F4/80^+$ and $Cd11b^+$ are shown in green.

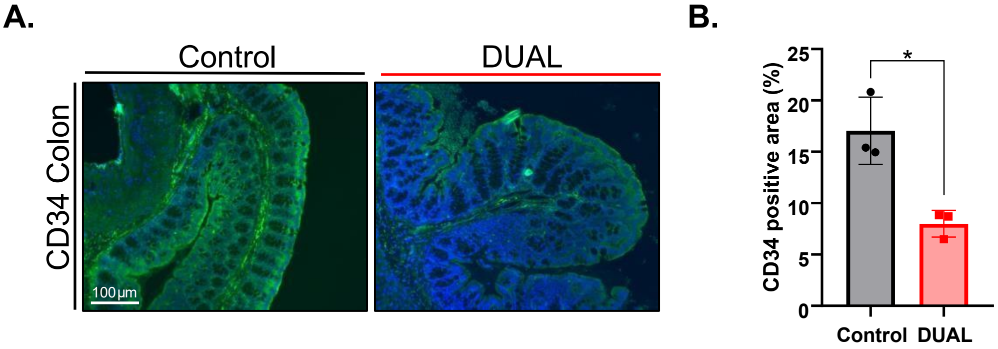

Suppl. Fig. 10. Disrupted GVB in colon of DUAL-fed mice. A. IF staining CD34 in colon. **B.** Positive stained area of CD34 was calculated respectively using Image J software (n=3).

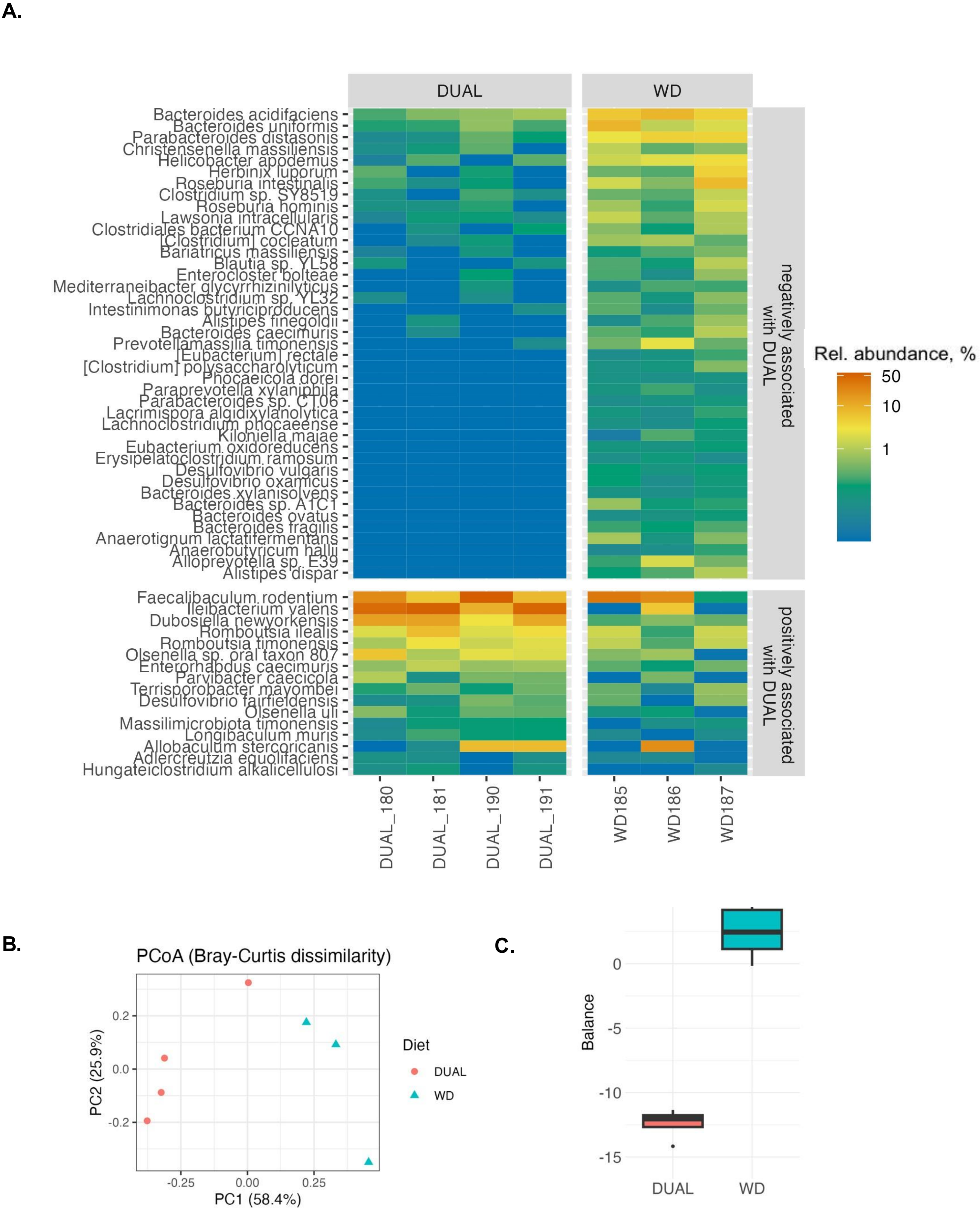

Suppl. Fig. 11. 16S rRNA gene amplicon profiles in DUAL vs. WD mice. ***A.*** *Bacterial taxa of the Nearest Balance (NB) analysis associated with DUAL as compared to the WD mice.* ***B.*** *Ordination plot for the microbiota composition based on Bray-Curtis dissimilarity.* ***C.*** *The values of the balance calculated for each sample compared between the groups.* (n=3-4).

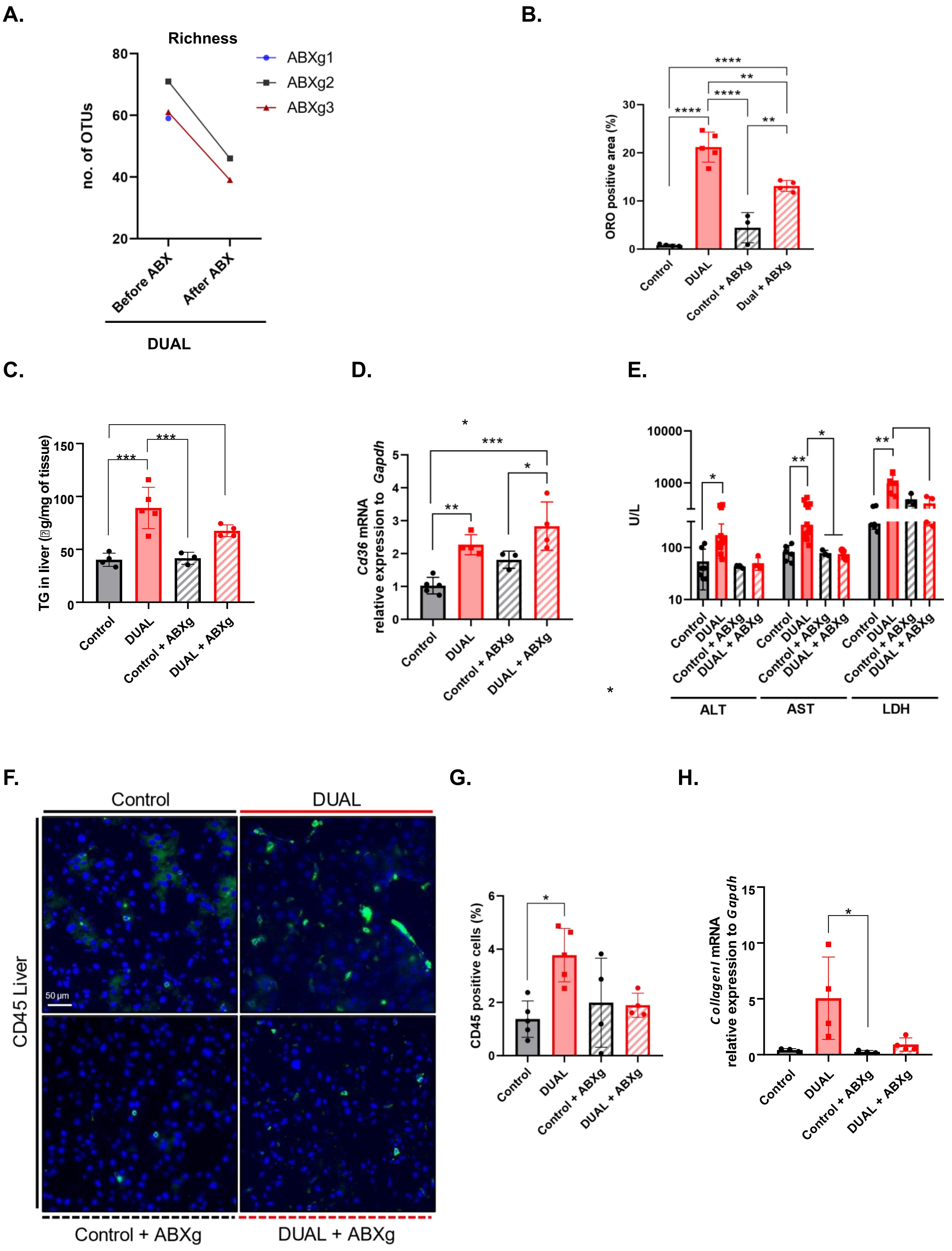
A.
Richness
ABXg1
ABXg2
ABXg3
no. of OTUs
80
60
40
20
Before ABX
After ABX
DUAL
B.
ORO positive area (%)
30
20
10
0
****
**
****
****
**
Control
DUAL
Control + ABXg
Dual + ABXg
C.
TG in liver (g/mg of tissue)
150
100
50
0
***
***
Control
DUAL
Control + ABXg
DUAL + ABXg
D.
Cd36 mRNA
relative expression to Gapdh
5
4
3
2
1
0
*
***
*
**
Control
DUAL
Control + ABXg
DUAL + ABXg
E.
U/L
10000
1000
100
10
*
**
*
**
Control
DUAL
Control + ABXg
DUAL + ABXg
ALT
AST
LDH
F.
Control
DUAL
CD45 Liver
50 µm
Control + ABXg
DUAL + ABXg
G.
CD45 positive cells (%)
6
4
2
0
*
Control
DUAL
Control + ABXg
DUAL + ABXg
H.
CollagenI mRNA
relative expression to Gapdh
15
10
5
0
*
Control
DUAL
Control + ABXg
DUAL + ABXg

Suppl. Fig. 12. Comprehensive depletion of the majority of gastrointestinal microflora and associated phenotype in DUAL mice after AIMD by oral gavage. **A.** OTU numbers in individual DUAL mice before and after antibiotic (ABX) treatment, showing a marked reduction following ABX administration (ABXg1 amplification was not possible after antibiotic treatment). **B.** Quantification of ORO-stained area (%) (n=3-4). **C.** Quantification of hepatic TG (µg/mg liver) (n=3-5). **D.** *Cd36* mRNA relative expression to *Gapdh* analysed by RT-qPCR in the liver (n=3-5). **E.** ALT, AST and LDH (U/L) levels in serum after 12 h fasting. (n=4-5). **F.** Representative CD45 IF stained liver sections. **G.** Quantification CD45-positive cells in the liver (%) using ImageJ software. (n=4-5). **H.** *CollagenI* mRNA relative expression to *Gapdh* in the liver (n=3-4).

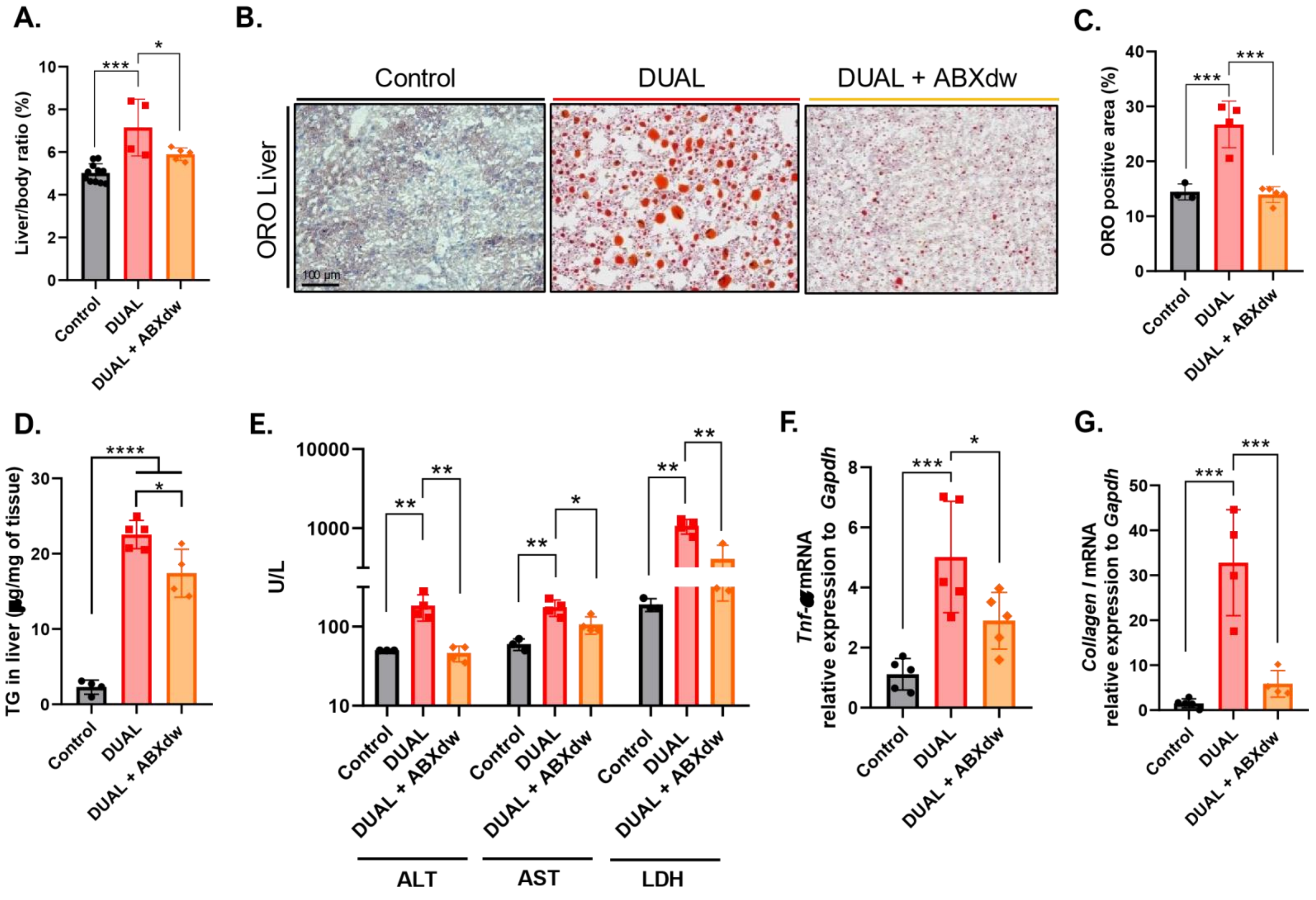

Suppl. Fig. 13. Attenuation of steatohepatitis in DUAL fed mice after antibiotic administration in drinking water. A. Liver/body weight ratio (%) (n=4-8). **B.** Illustrative ORO-stained liver sections. **C.** Quantification of ORO-stained area (%) (n=3-5). **D.** Quantification of hepatic TG (μg/mg liver) (n=4-5). **E.** ALT, AST and LDH (U/L) levels in serum after 12 h fasting. (n=4-5). **F-G.** *Tnf-α* and *Collagen I* mRNA relative expression to *Gapdh* analysed by RT-qPCR in the liver respectively (n=4-5).

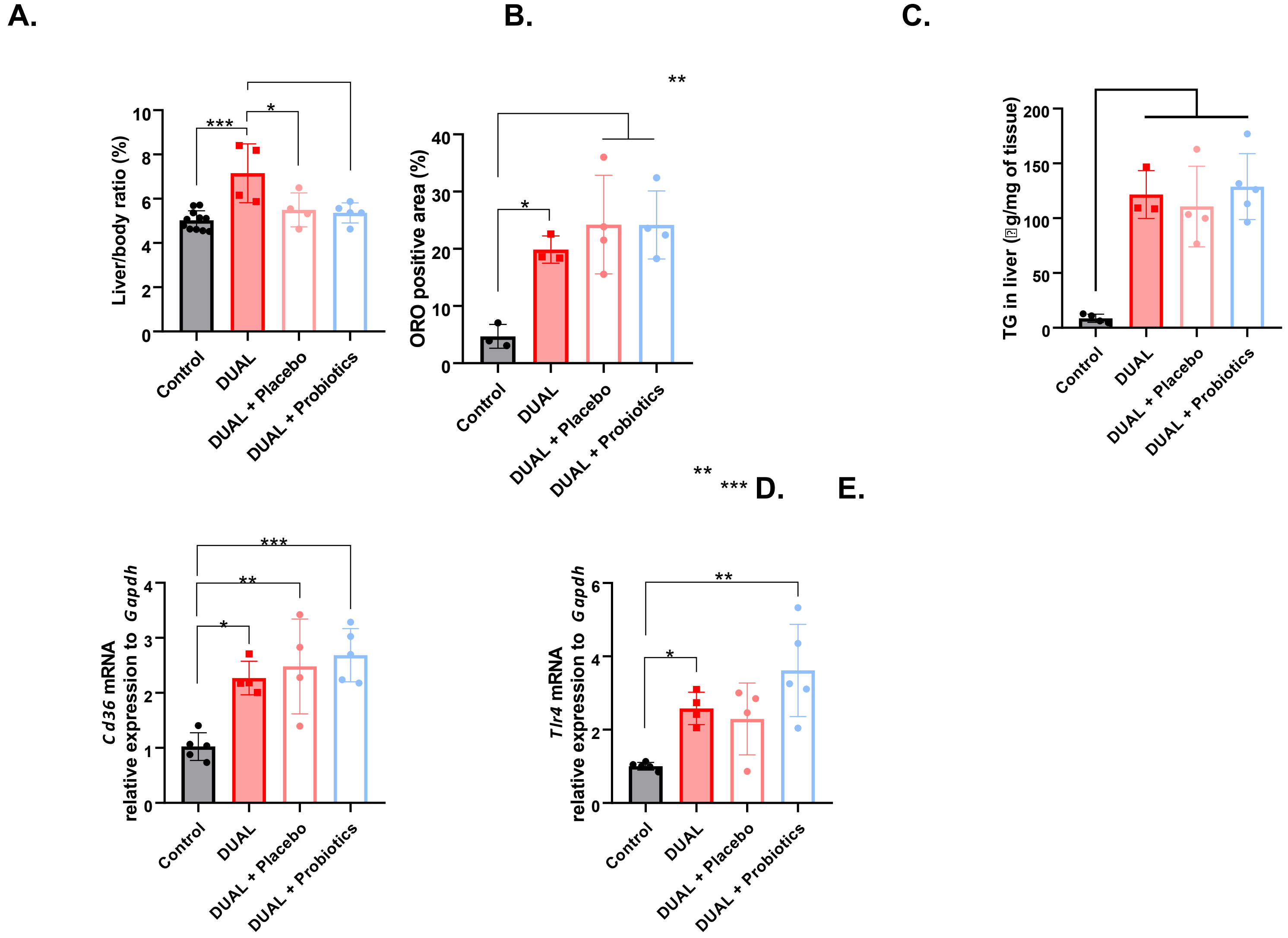

Suppl. Fig. 14. Hepatic steatosis and associated liver damage in DUAL mice treated with probiotics. A. Liver/body weight ratio after probiotic administration (%) (n=4-8). **B.** Quantification of ORO-stained area (%) (n=3-4). **C.** Quantification of hepatic TG (μg/mg liver) (n=3-5). **D.** *Cd36* mRNA relative expression to *Gapdh* analysed by RT-qPCR in the liver (n=4-5). **E.** Hepatic *Tlr4* mRNA relative expression to *Gapdh* (n=4-5).

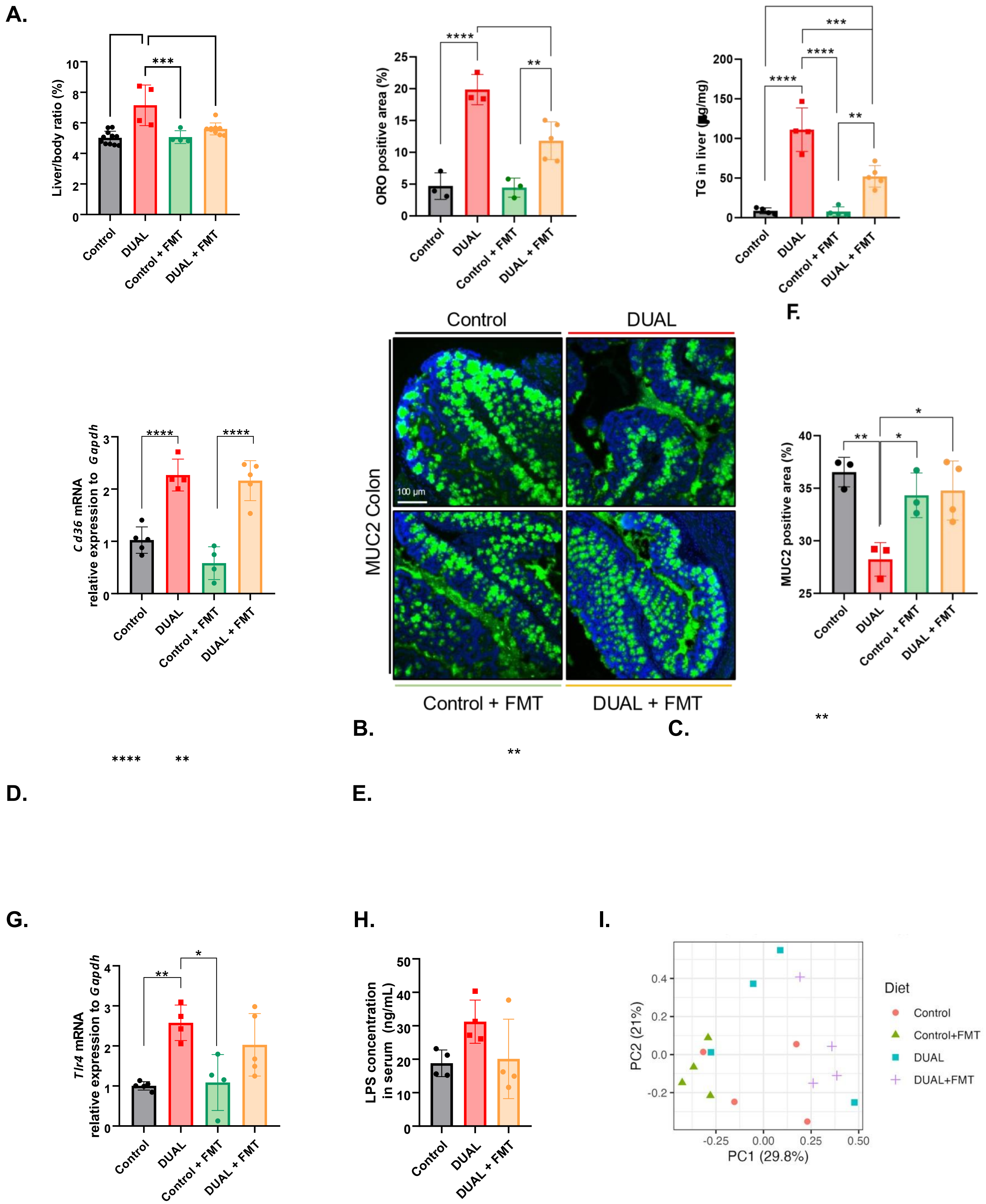
A.
Liver/body ratio (%)
***
Control
DUAL
Control + FMT
DUAL + FMT
ORO positive area (%)
****
**
TG in liver (μg/mg)
***
****
****
**
B.
Control
DUAL
MUC2 Colon
100 μm
Control + FMT
DUAL + FMT
C.
D.
Cd36 mRNA relative expression to Gapdh
****
****
E.
F.
MUC2 positive area (%)
**
*
*
G.
Tlr4 mRNA relative expression to Gapdh
**
*
H.
LPS concentration in serum (ng/mL)
I.
PC2 (21%)
PC1 (29.8%)
Diet
Control
Control+FMT
DUAL
DUAL+FMT

Suppl. Fig. 15. Hepatic and intestinal alterations in liver and gut of DUAL -fed mice after FMT. **A.** Liver/body weight ratio (%) after FMT (n=4-8). **B.** Quantification of ORO-stained area (%) (n=3-4). **C.** Quantification of hepatic TG (µg/mg liver) (n=4-5). **D.** *Cd36* mRNA relative expression to *Gapdh* analysed by RT-qPCR in the liver (n=4-5). **E.** IF MUC2 staining in colon. **F.** Quantification of positive MUC2 stained area (%) by Image J software. (n=3-4). **G.** *Tlr4* mRNA relative expression to *Gapdh* analyzed by RT-qPCR in the liver (n=4-5). **H.** LPS (ng/mL) in serum (n=4). **I.** General microbiome dynamics according to Bray-Curtis beta diversity. “Control” – healthy donors, “DUAL” – DUAL mice, “DUAL+FMT” – the latter group having received FMT from the former donors, “Control+FMT” – other healthy mice having received FMT from yet other healthy mice.

A.

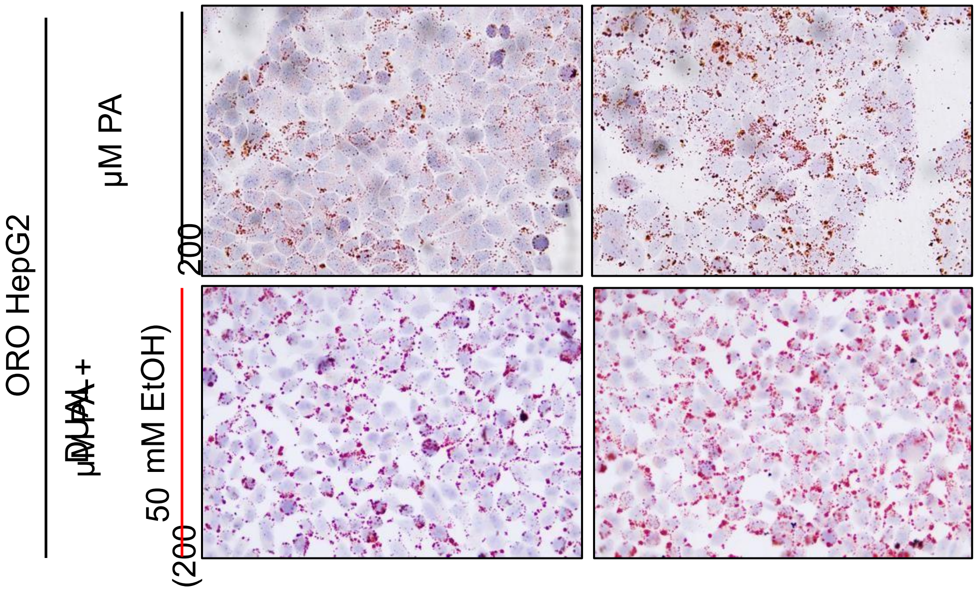

B.

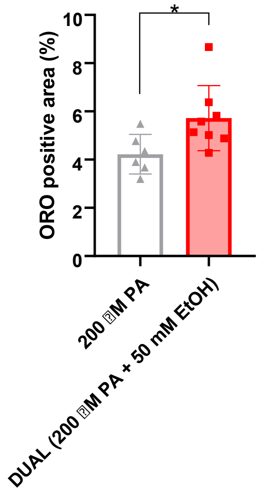

C.

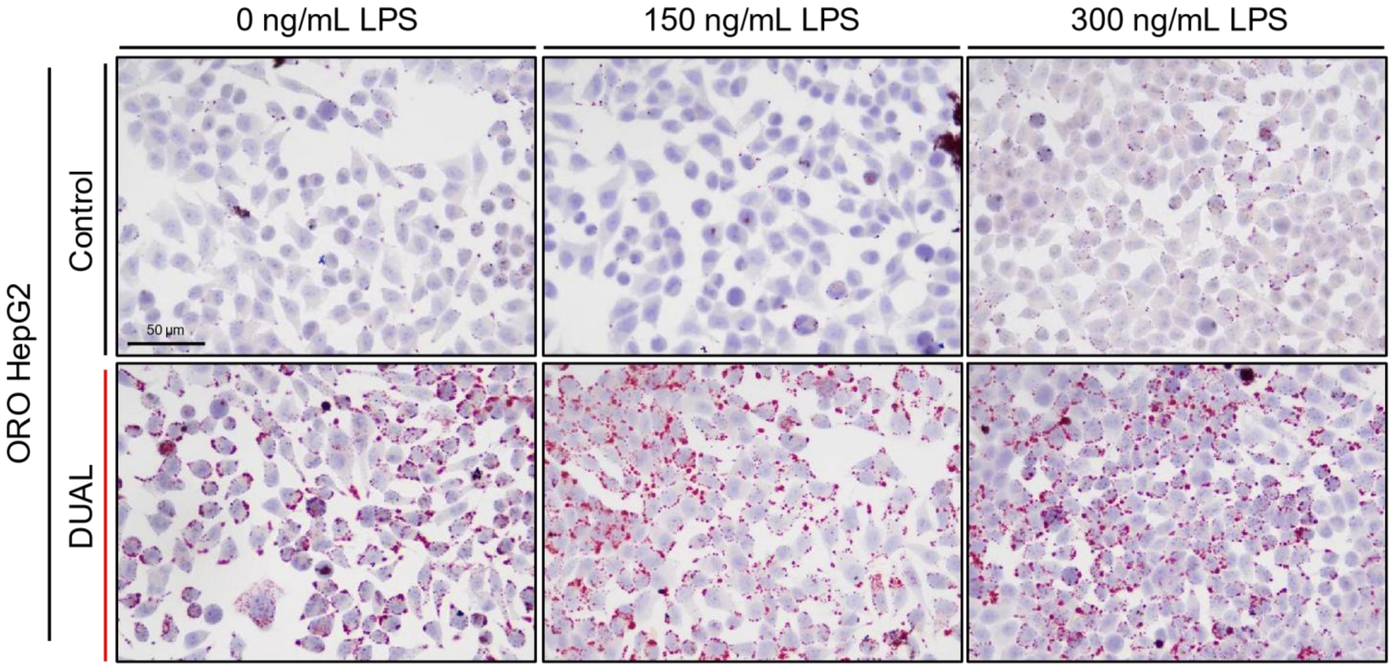

D.

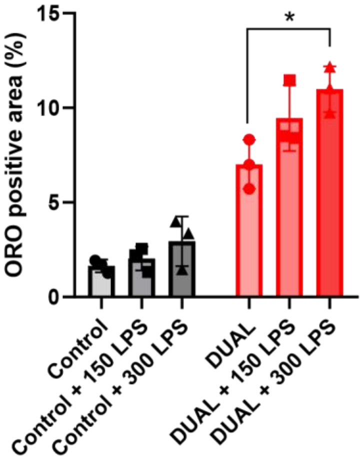

E.

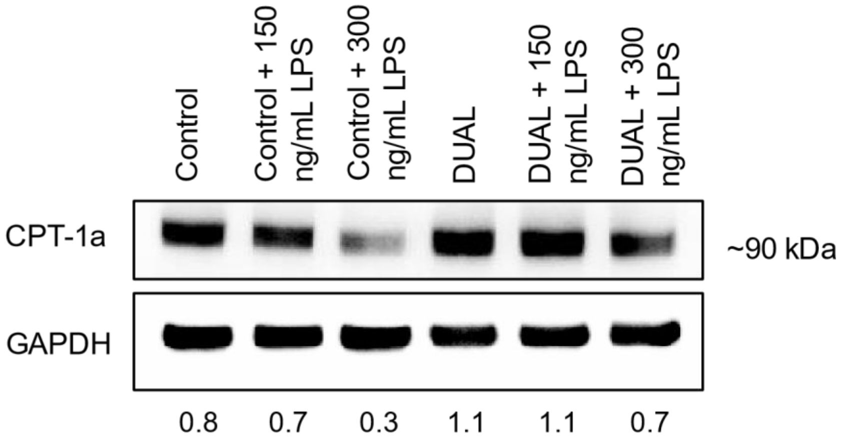

70 kDa

Suppl. Fig. 16. In vitro effects of PA, DUAL and DUAL+LPS treatment in HepG2 cells. A. Illustrative ORO-stained HepG2 cells treated with 200 µM PA or DUAL (200 µM PA + 50 mM EtOH). **B.** Quantification of ORO-stained area (%) (n=1). **C.** Illustrative ORO-stained HepG2 cells. **D.** Quantification of ORO-stained area (%) (n=3). **E.** CPT-1a WB in HepG2 cells. HSC70 was used as a loading control. CPT-1a/GAPDH protein level ratio was calculated.

A. ZEBRAFISH

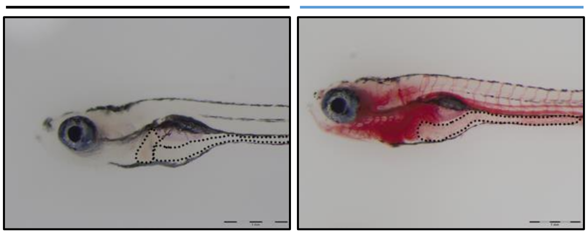

B. ZEBRAFISH

Ctrl EtOH/HFHC

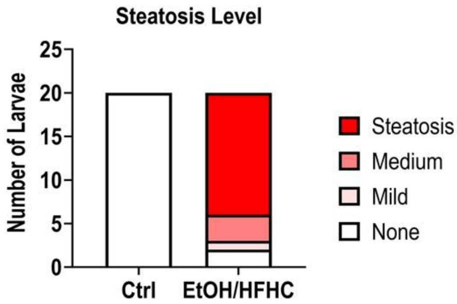

C. ZEBRAFISH

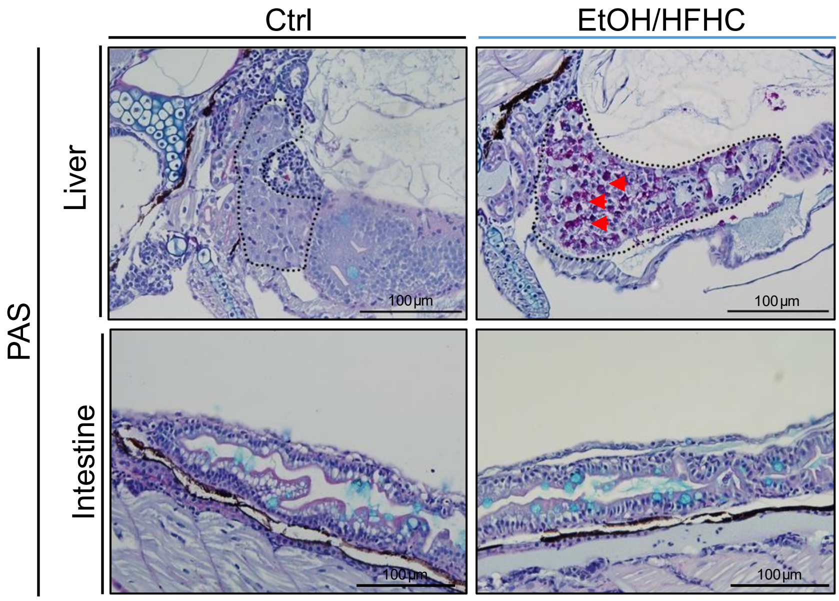

Suppl. Fig. 17. Diet-induced hepatomegaly and hepatic steatosis in zebrafish larvae. A. ORO staining images of 9 dpf zebrafish larvae fed with Ctrl diet or 0.5%EtOH/HFHC diet. White dotted lines outline larvae livers. Scale bar: 500 µm. **B.** Quantification of steatosis based on ORO staining. Levels were specified as none (no steatosis), mild, medium and steatosis. (n=30) **C.** Alcian blue/PAS staining on paraffin sections of 9 dpf larvae fed with Ctrl or EtOH/HFHC. Black dotted line outlines liver. Red arrows point ballooned hepatocytes. In the intestine blue turquoise areas represent mucins, goblet cells; blue-purple area nuclei (n=5). Scale bar: 100 µm.

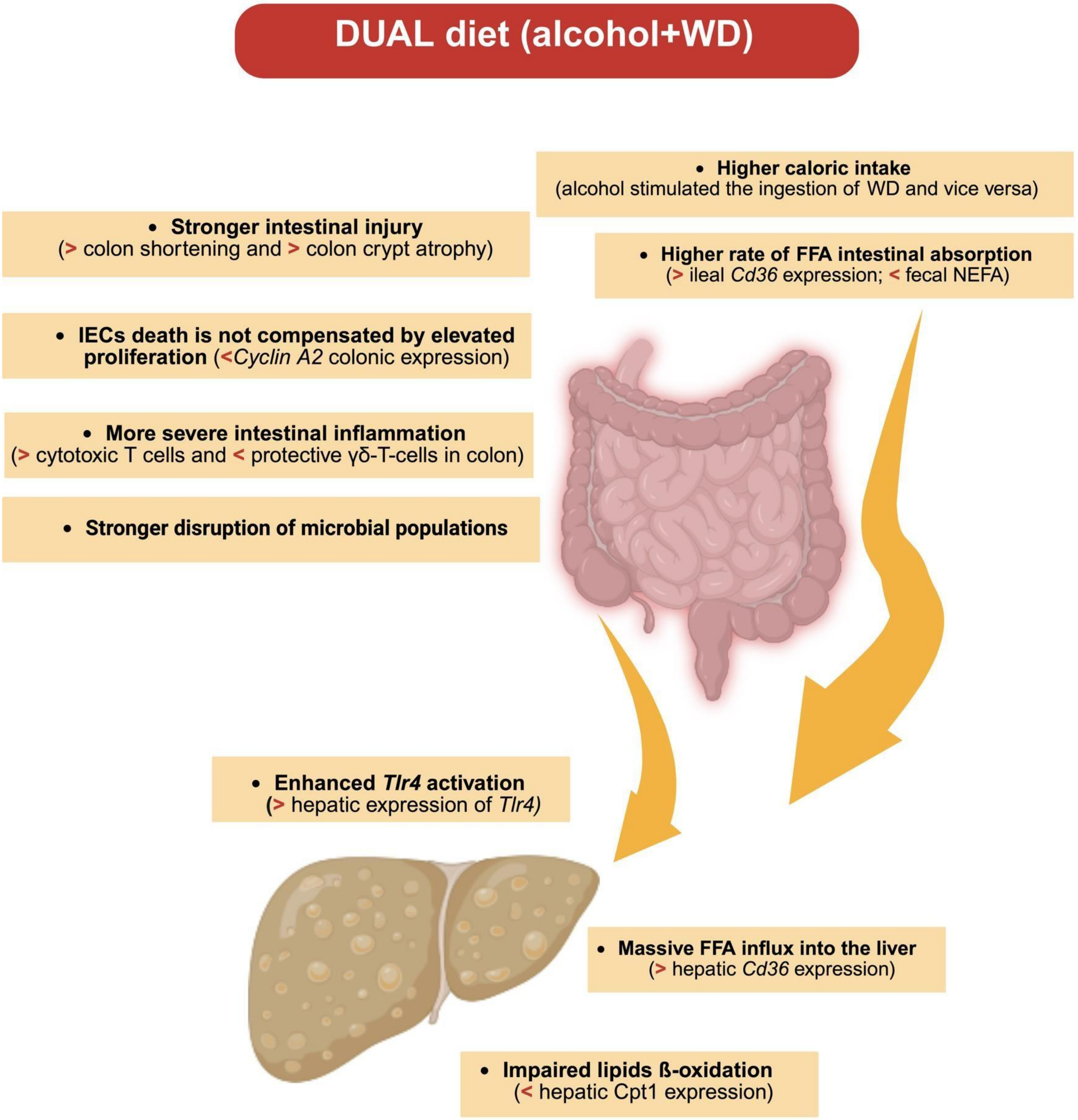

Suppl. Fig. 18. (A) A schematic overview of major differences observed at various levels of the gut-liver axis in DUAL mice compared with WD-fed mice, which altogether contribute to the more pronounced steatohepatitis and intestinal inflammation (Created in https://BioRender.com)

A.

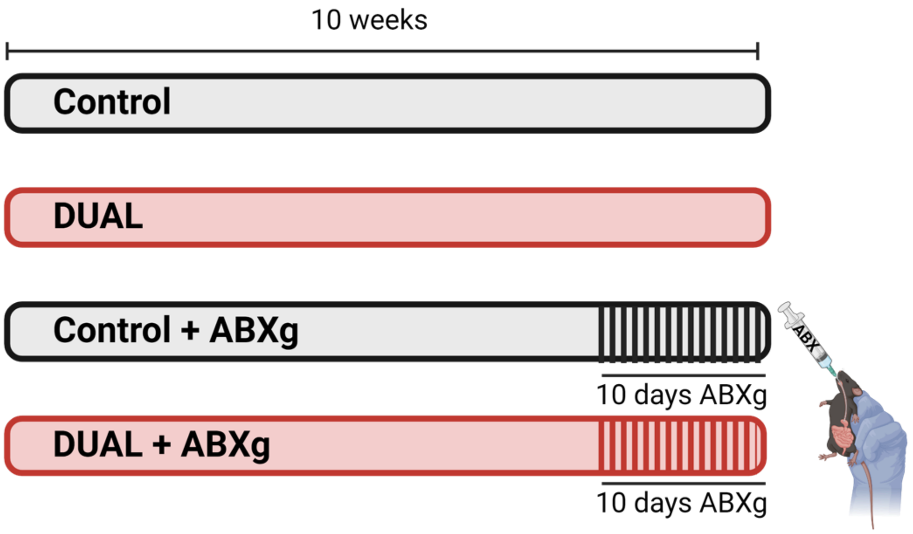

B.

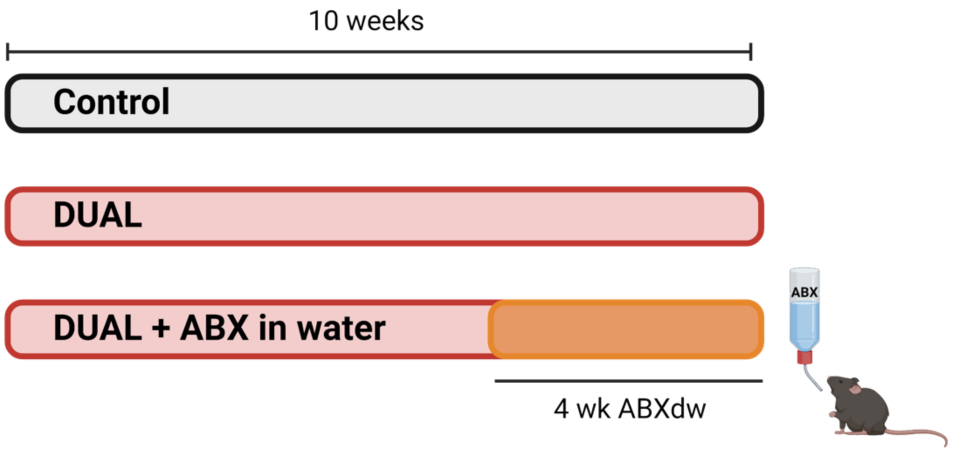

Suppl. Fig. 19. Experimental design of antibiotic (ABX) administration. Mice received DUAL or control diet for 10 weeks; **A.** During the last 10 days of the feeding mice received antibiotic cocktail by oral gavage every 24 hours (DUAL+ABXg or Control+ABXg). **B.** A broad-spectrum cocktail of antibiotics was added to drinking water during the final 4 weeks of DUAL feeding (DUAL+ABXdw) **(Created in https://BioRender.com).**

Suppl. Fig. 20. Experimental design of probiotic/placebo supplementation. A. Mice received

A.

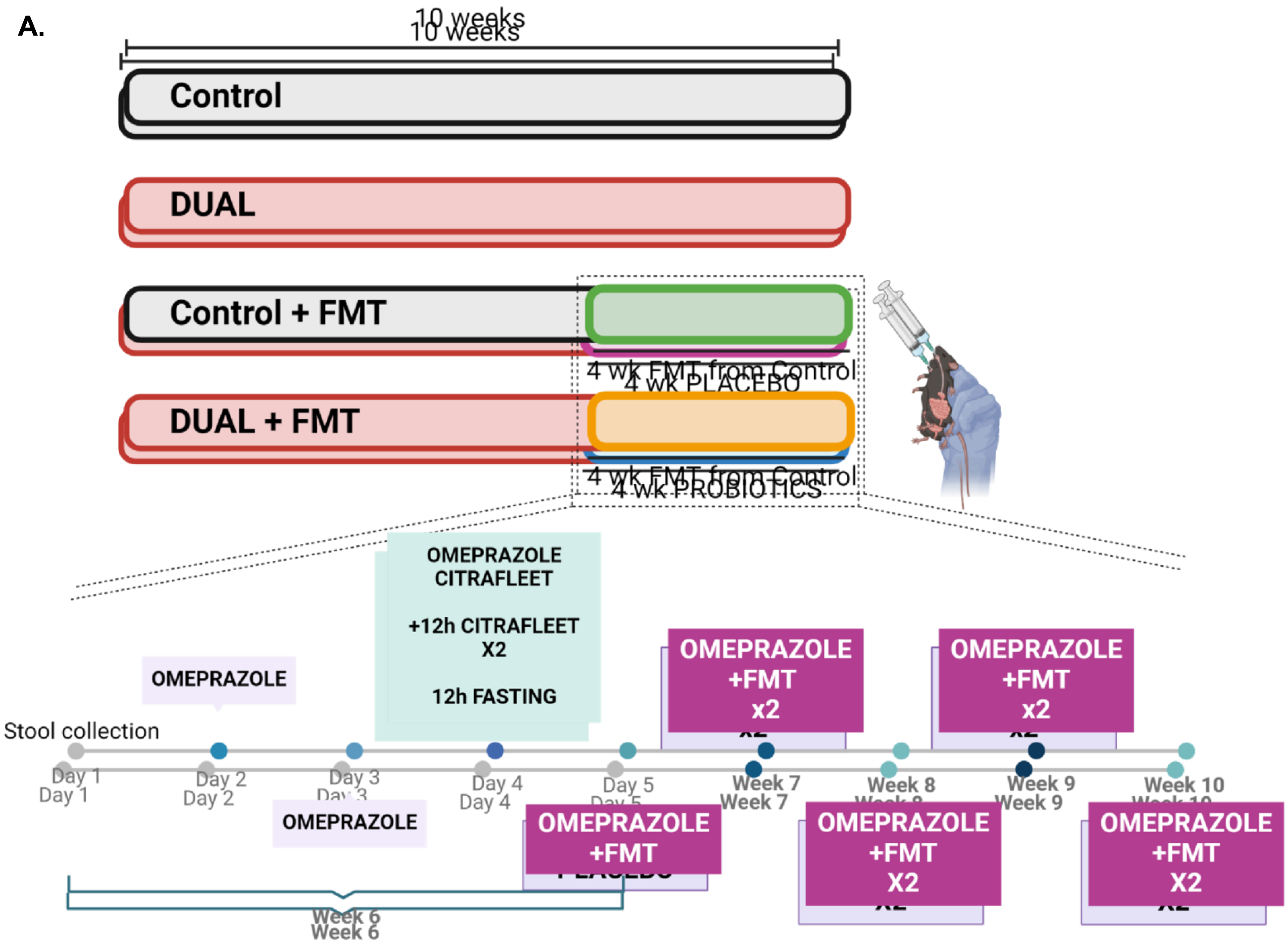

DUAL or control diet for 10 weeks. During the last 4 weeks of DUAL feeding probiotic/placebo was gavaged to the animals twice a week until the experimental endpoint **(Created in https://BioRender.com).**

Suppl. Fig. 21. Detailed experimental scheme of FMT. A. Mice received DUAL or control diet for 10 weeks FMT was performed with fresh stool pellets from healthy donors twice a week by oral gavage during the last 4 weeks of feeding period **(Created in https://BioRender.com).**
